# Supplementary figures and images for: A cleaved METTL3 potentiates the METTL3–WTAP interaction and breast cancer progression
Source: eLife. 2023 Aug 17;12:RP87283. doi: 10.7554/eLife.87283 (PMC10435237; doi:10.7554/eLife.87283)

**Figure 1-source data:** Unedited western blot pictures for figure 1.

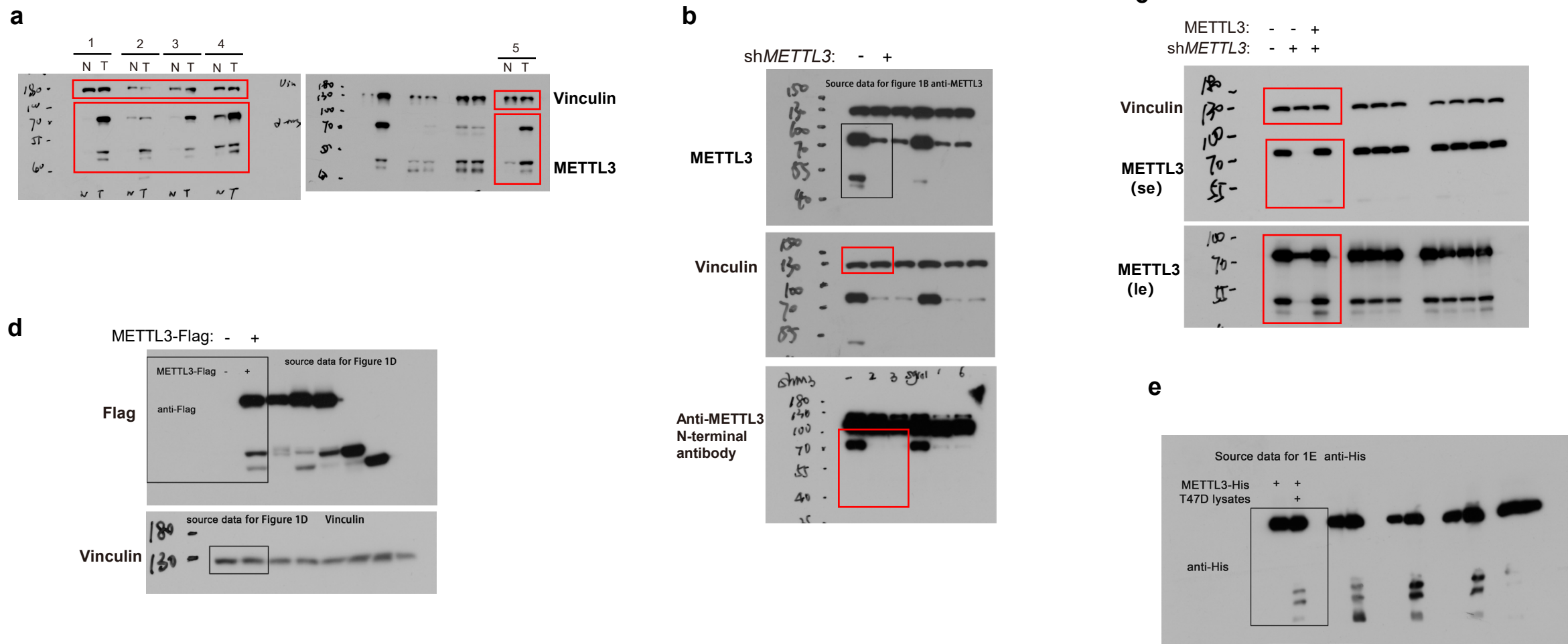

Supplement: Figure 1—source data 1. [file elife-87283-fig1-data1.pdf]

**Figure 2-source data-1:** Unedited western blot pictures for figure 2.

**b**

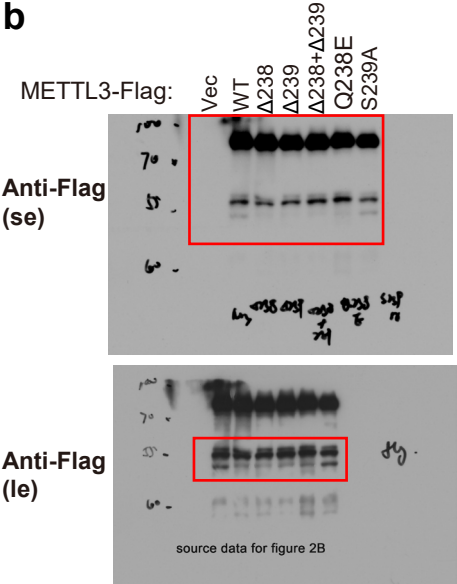

**d**

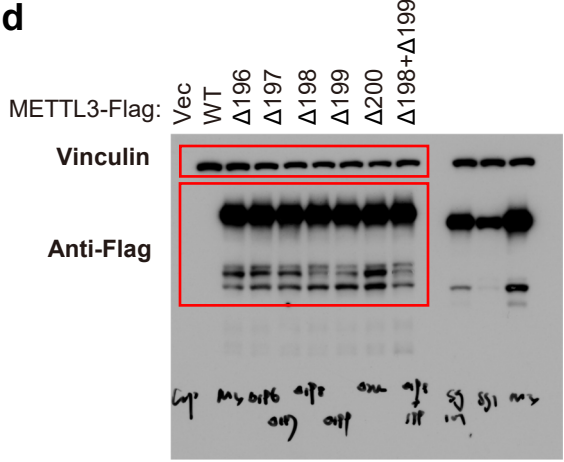

**e**

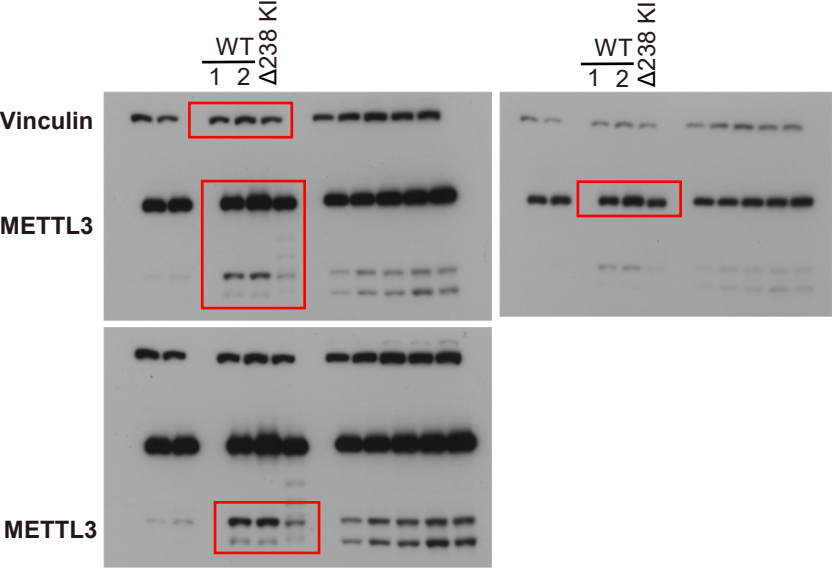

**f**

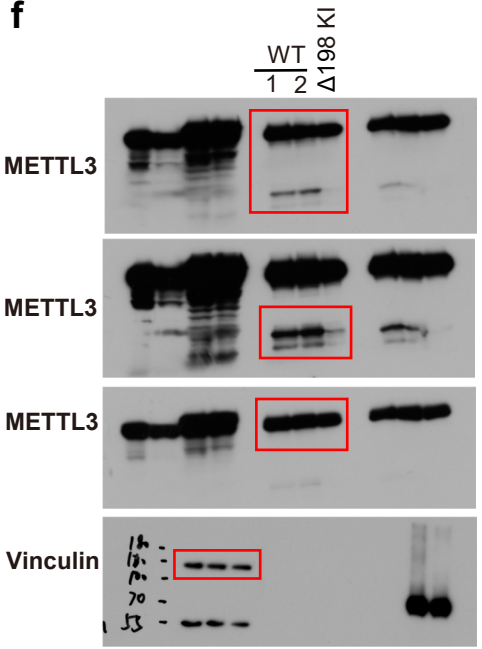

**g**

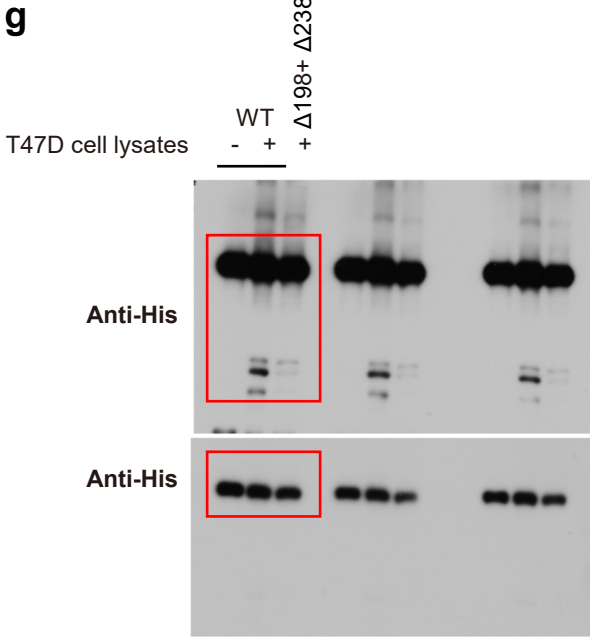

**h**

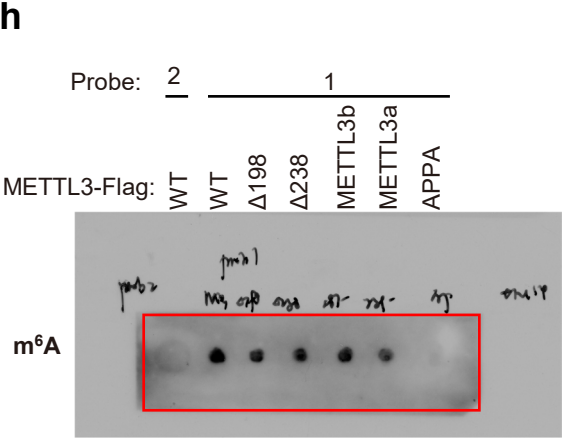

**j**

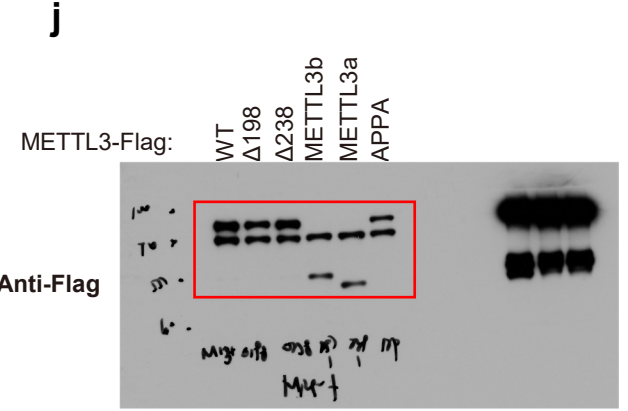

Supplement: Figure 2—source data 1. [file elife-87283-fig2-data1.pdf]

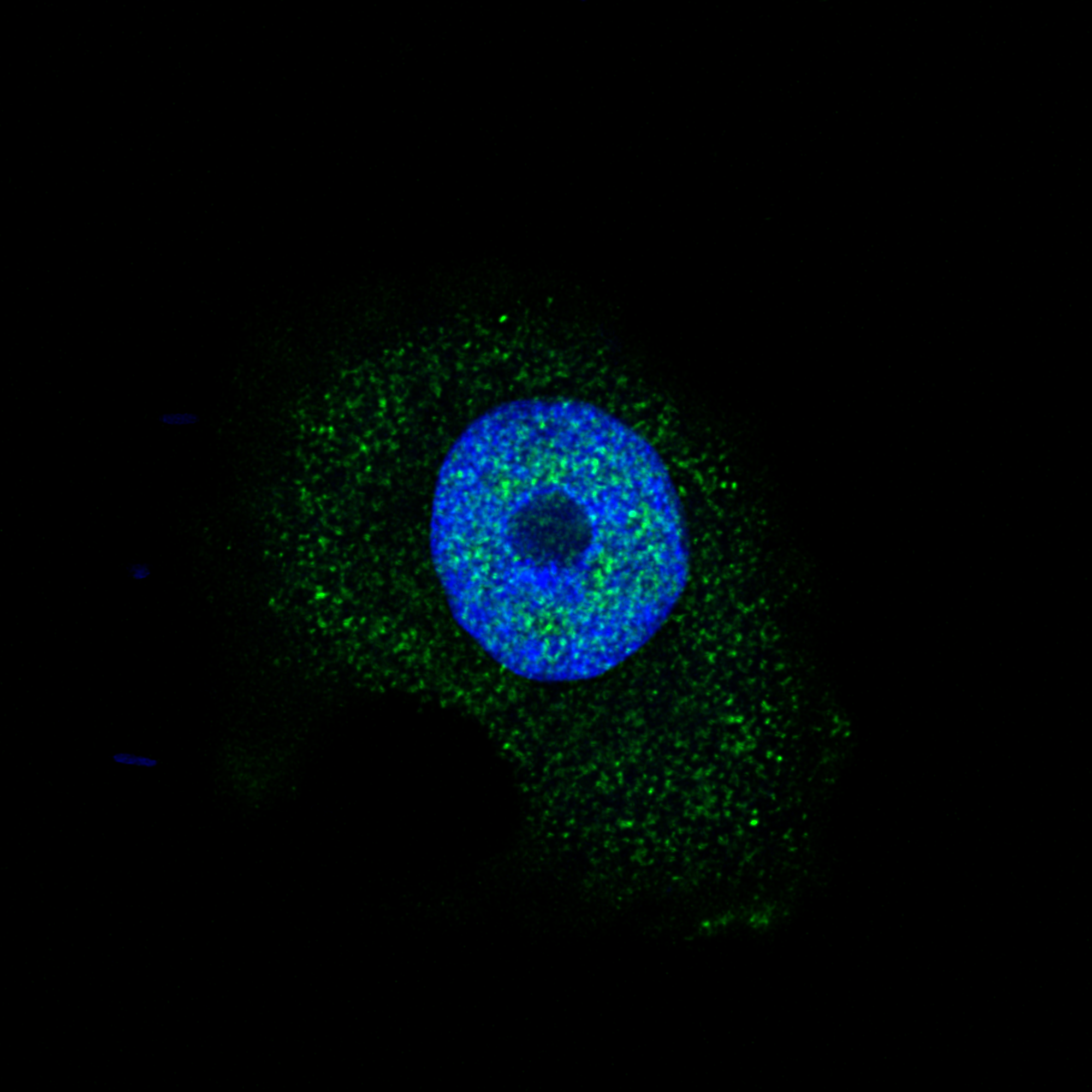

Supplement: Figure 2—figure supplement 1—source data 1. [file elife-87283-fig2-figsupp1-data1.zip › Figure 2-Figure supplement 1-source data/M3sg1-197-580-Flag Merged.tif]

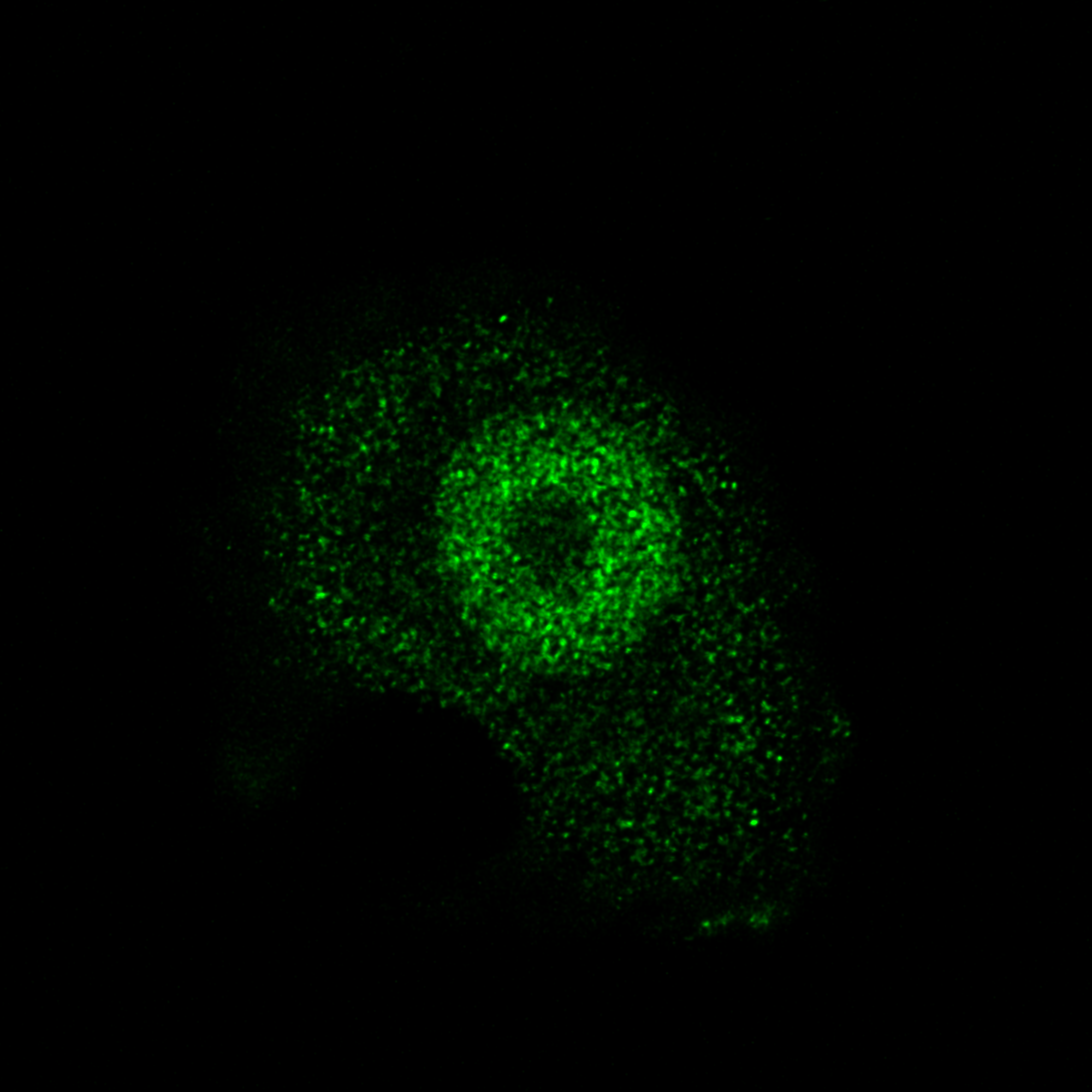

Supplement: Figure 2—figure supplement 1—source data 1. [file elife-87283-fig2-figsupp1-data1.zip › Figure 2-Figure supplement 1-source data/M3sg1-197-580-Flag_488.tif]

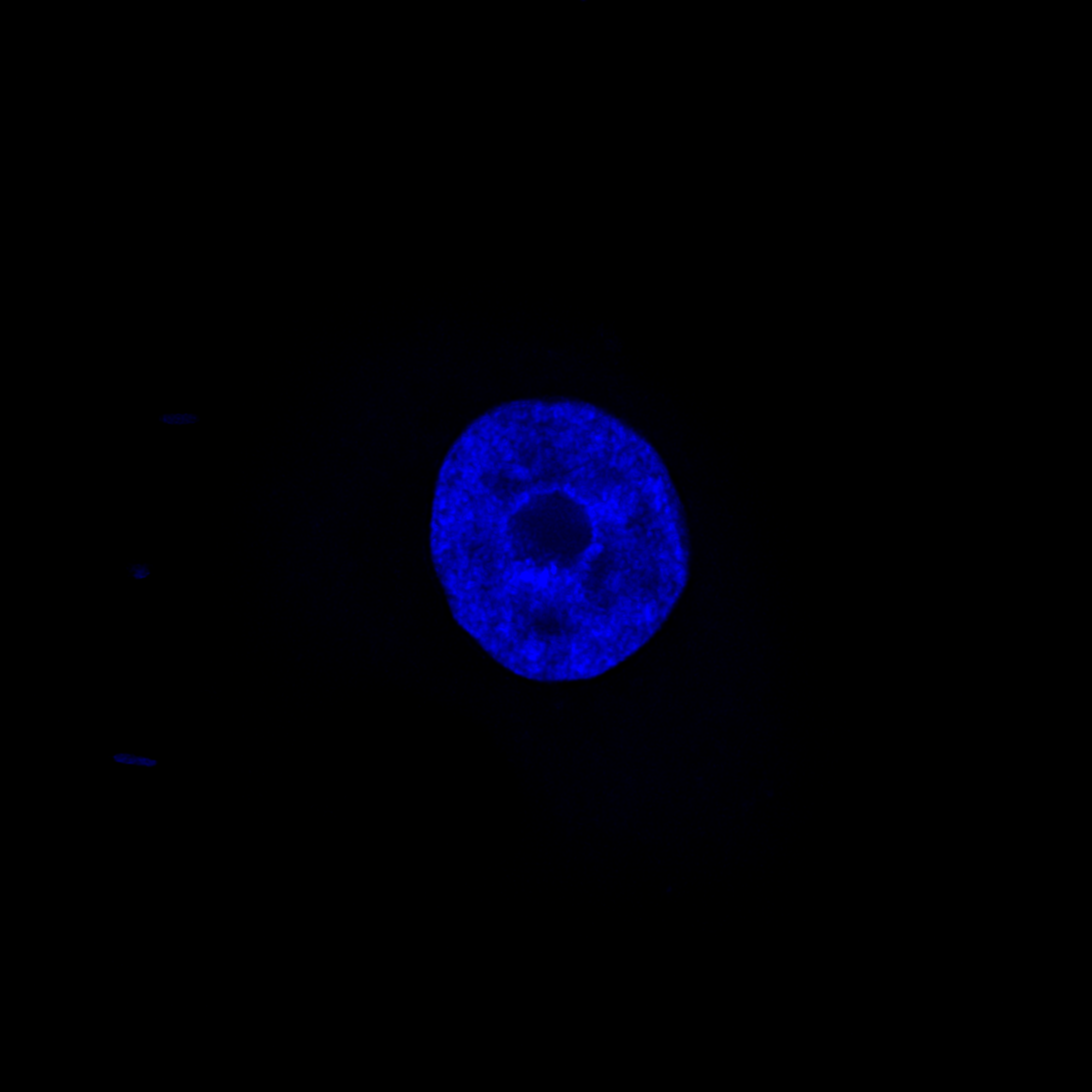

Supplement: Figure 2—figure supplement 1—source data 1. [file elife-87283-fig2-figsupp1-data1.zip › Figure 2-Figure supplement 1-source data/M3sg1-197-580-Flag_DAPI.tif]

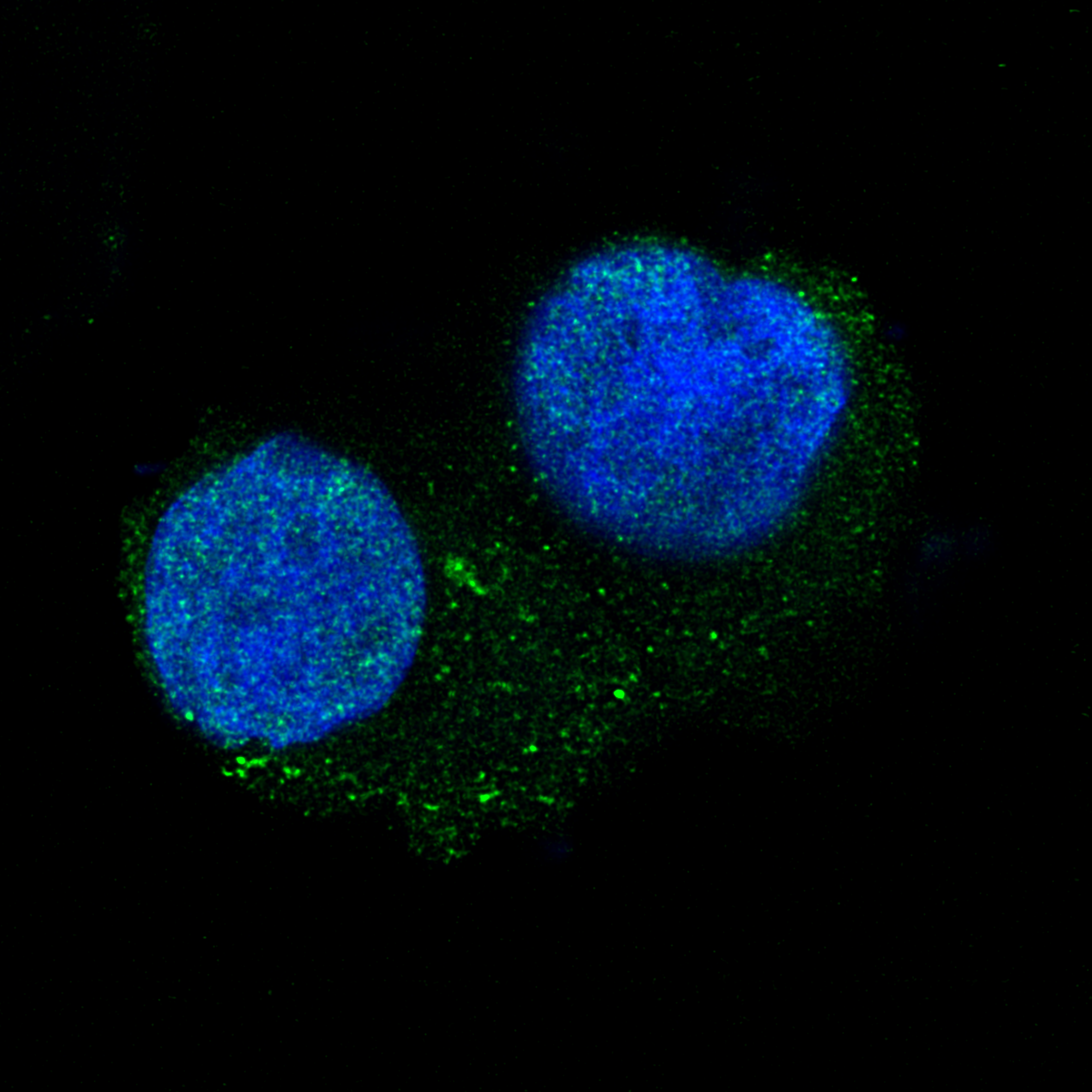

Supplement: Figure 2—figure supplement 1—source data 1. [file elife-87283-fig2-figsupp1-data1.zip › Figure 2-Figure supplement 1-source data/M3sg1-239-580-Flag Merged.tif]

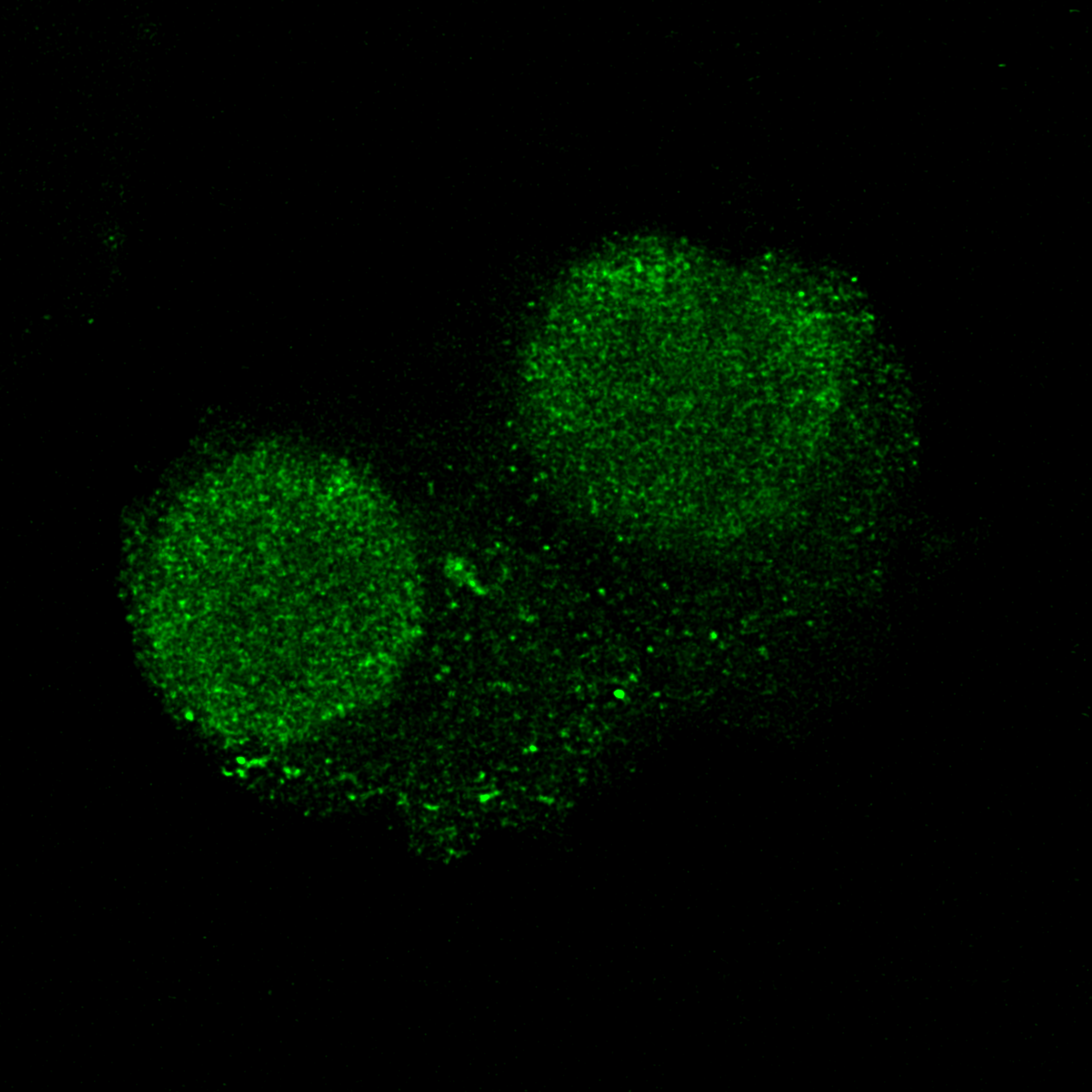

Supplement: Figure 2—figure supplement 1—source data 1. [file elife-87283-fig2-figsupp1-data1.zip › Figure 2-Figure supplement 1-source data/M3sg1-239-580-Flag_488.tif]

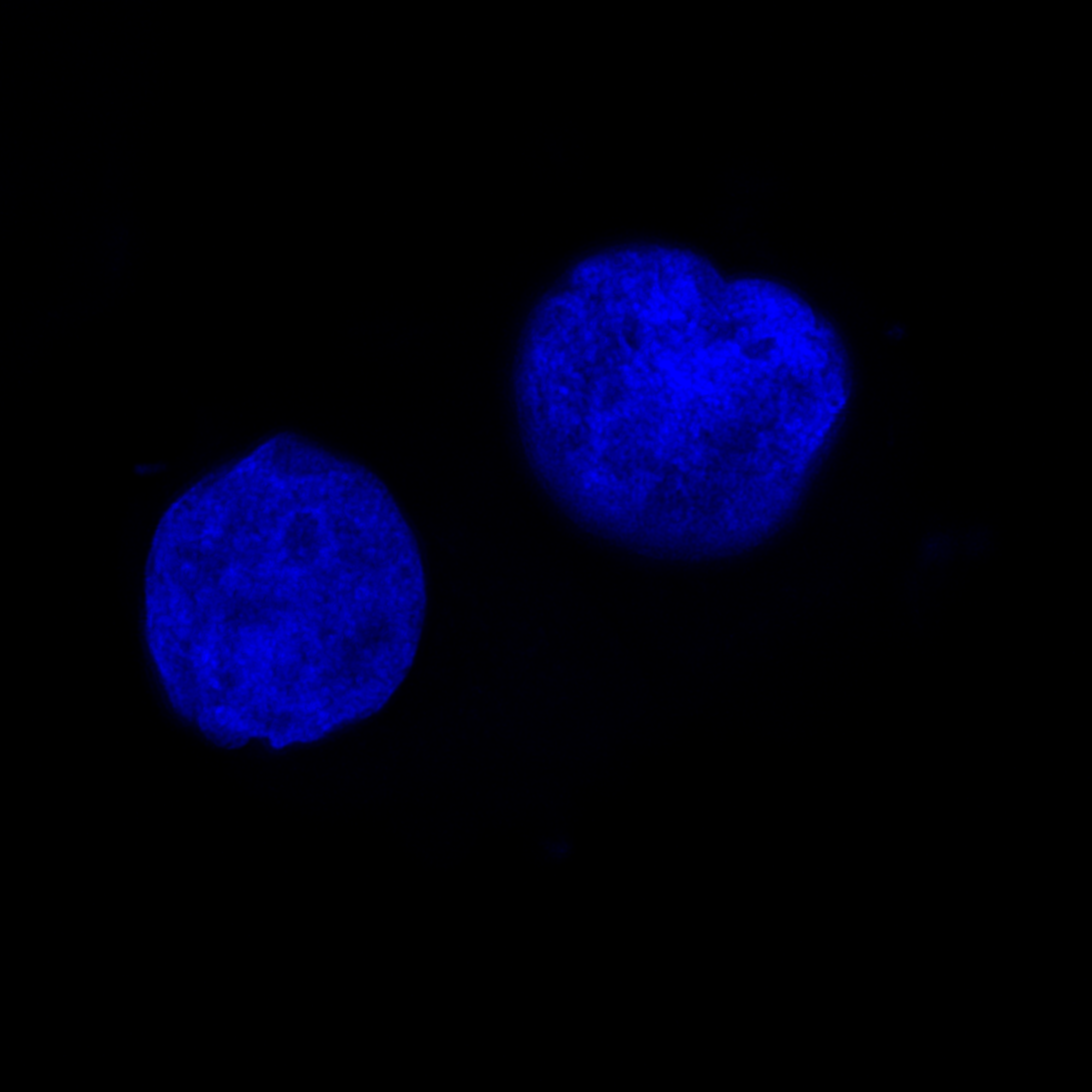

Supplement: Figure 2—figure supplement 1—source data 1. [file elife-87283-fig2-figsupp1-data1.zip › Figure 2-Figure supplement 1-source data/M3sg1-239-580-Flag_DAPI.tif]

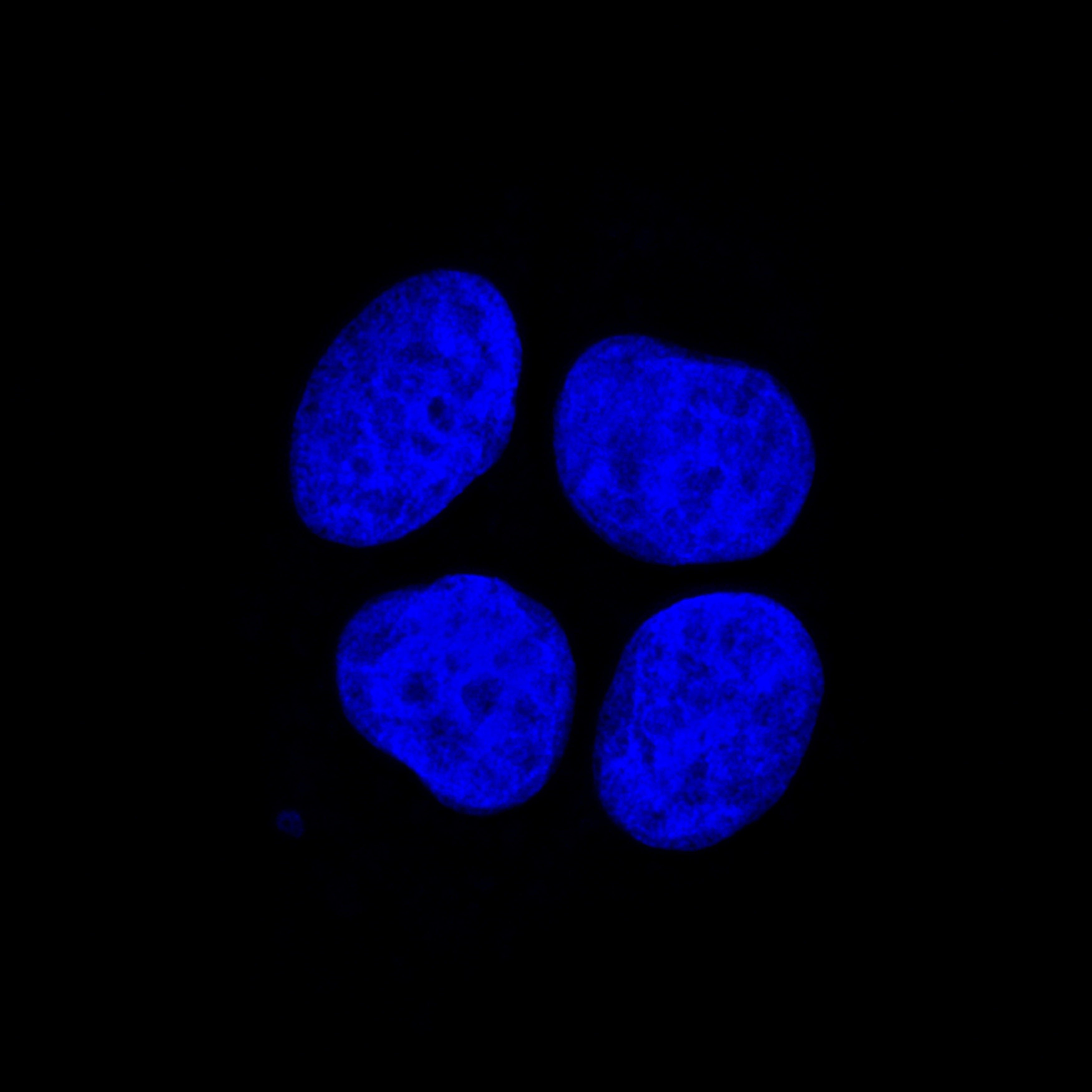

Supplement: Figure 2—figure supplement 1—source data 1. [file elife-87283-fig2-figsupp1-data1.zip › Figure 2-Figure supplement 1-source data/M3sg1-delt198-Flag DAPI.tif]

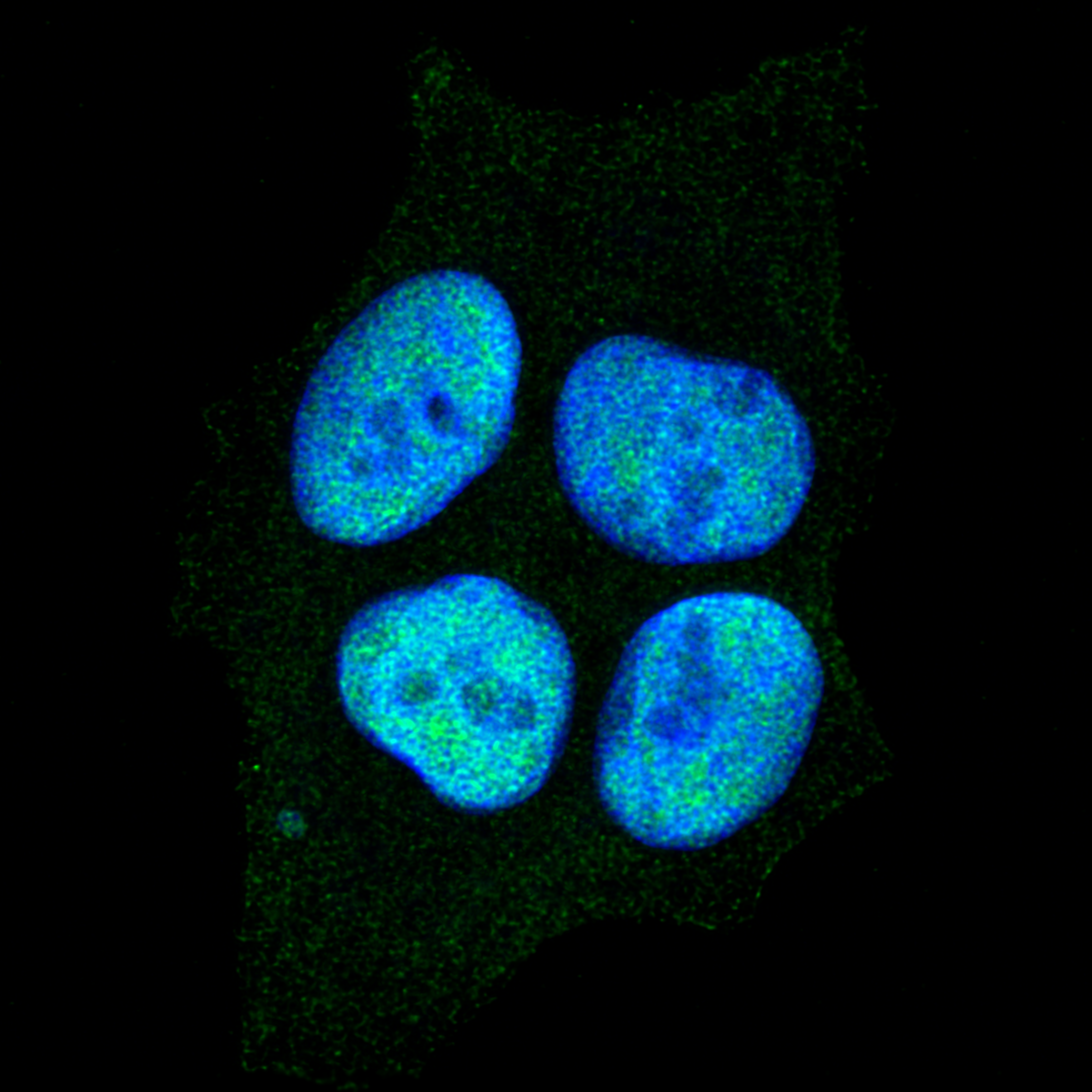

Supplement: Figure 2—figure supplement 1—source data 1. [file elife-87283-fig2-figsupp1-data1.zip › Figure 2-Figure supplement 1-source data/M3sg1-delt198-Flag Merged.tif]

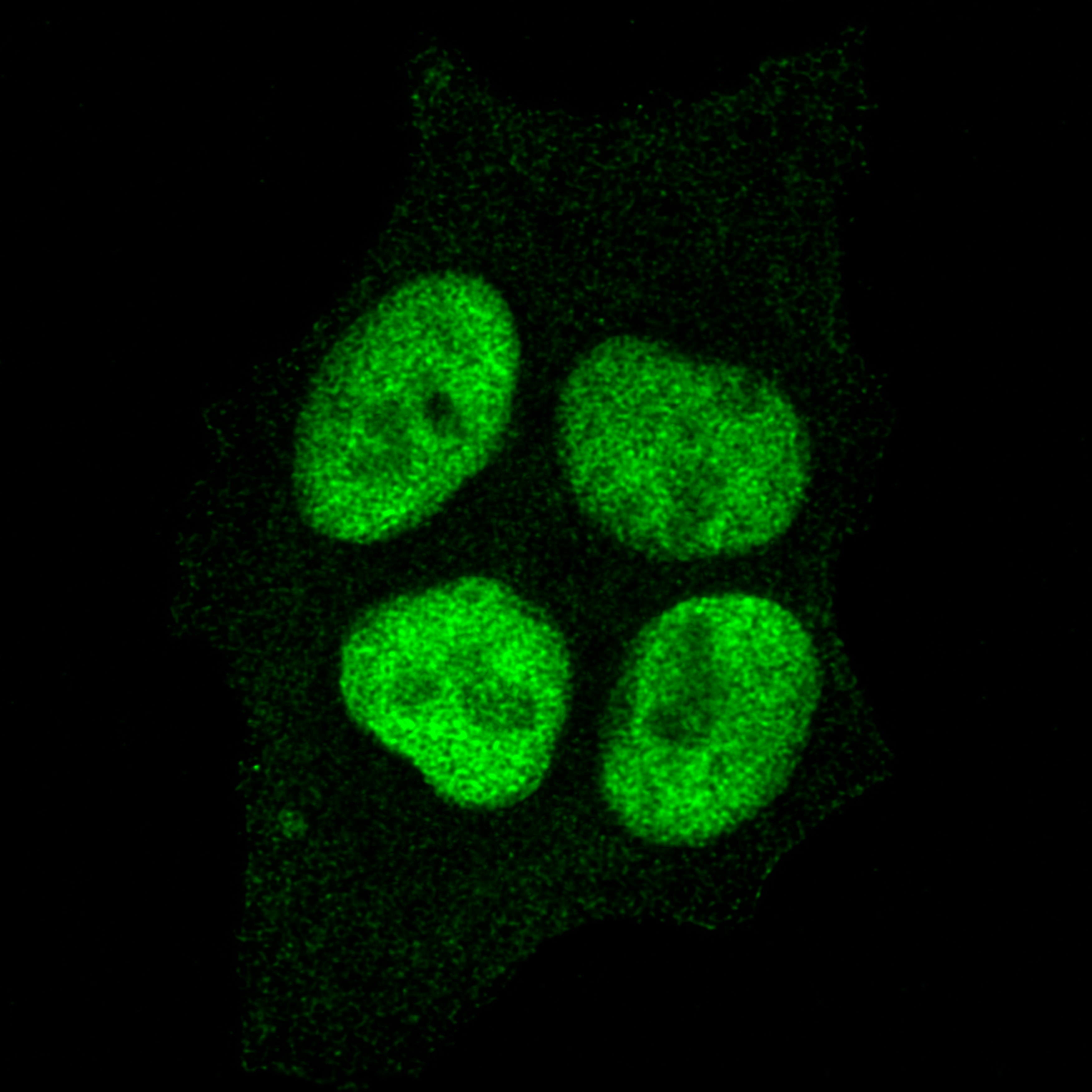

Supplement: Figure 2—figure supplement 1—source data 1. [file elife-87283-fig2-figsupp1-data1.zip › Figure 2-Figure supplement 1-source data/M3sg1-delt198-Flag_488.tif]

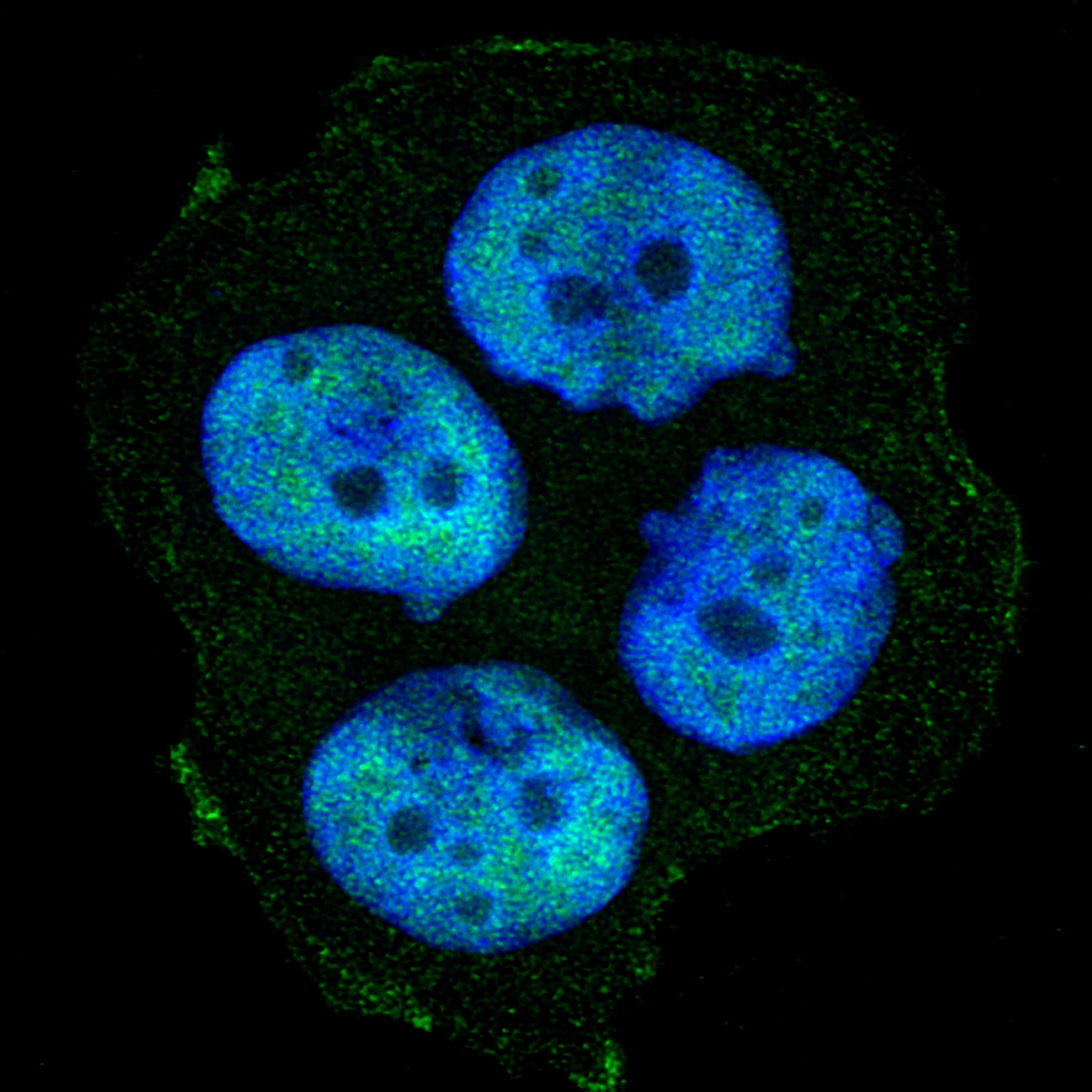

Supplement: Figure 2—figure supplement 1—source data 1. [file elife-87283-fig2-figsupp1-data1.zip › Figure 2-Figure supplement 1-source data/M3sg1-delt238-Flag merged.tif]

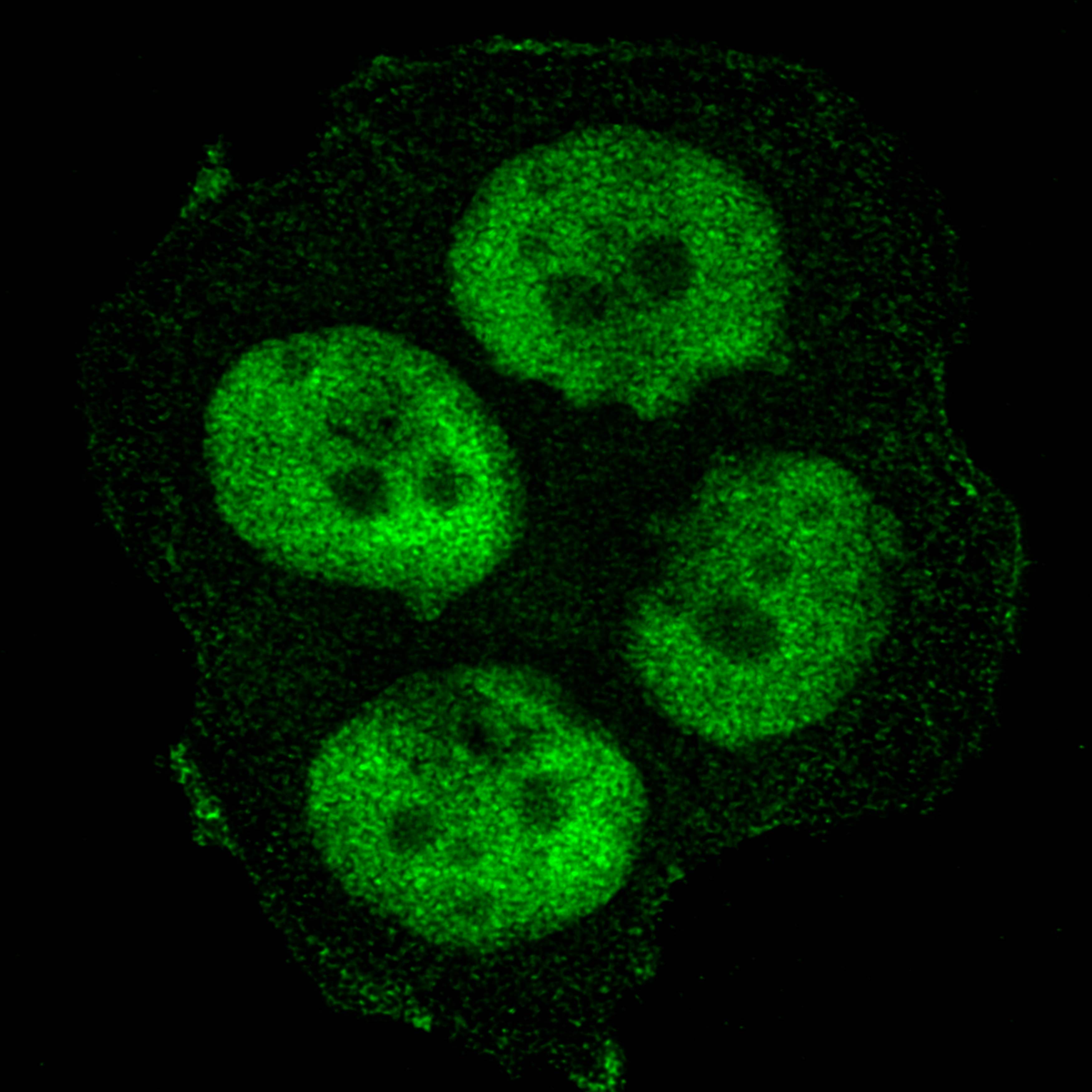

Supplement: Figure 2—figure supplement 1—source data 1. [file elife-87283-fig2-figsupp1-data1.zip › Figure 2-Figure supplement 1-source data/M3sg1-delt238-Flag_488.tif]

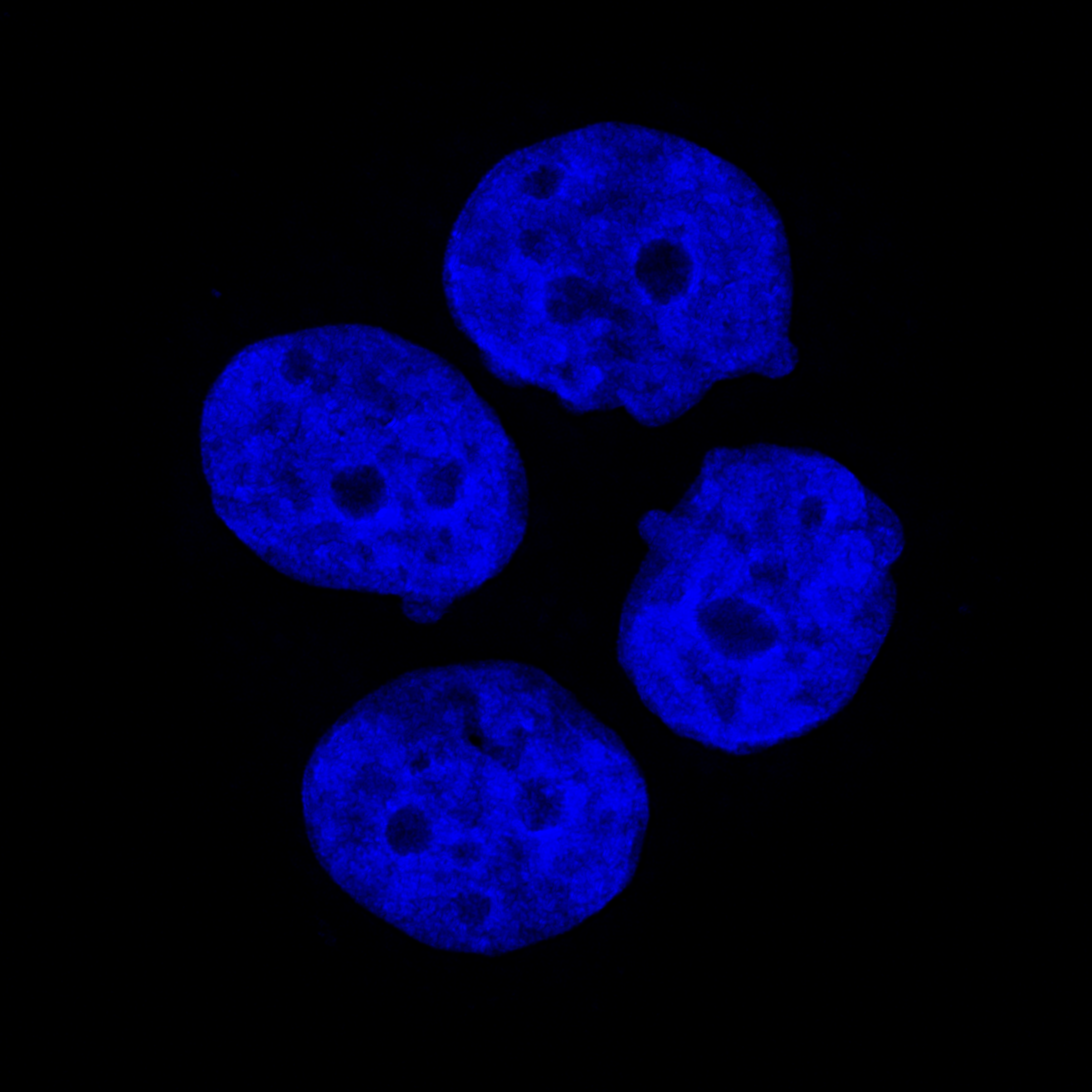

Supplement: Figure 2—figure supplement 1—source data 1. [file elife-87283-fig2-figsupp1-data1.zip › Figure 2-Figure supplement 1-source data/M3sg1-delt238-Flag_DAPI.tif]

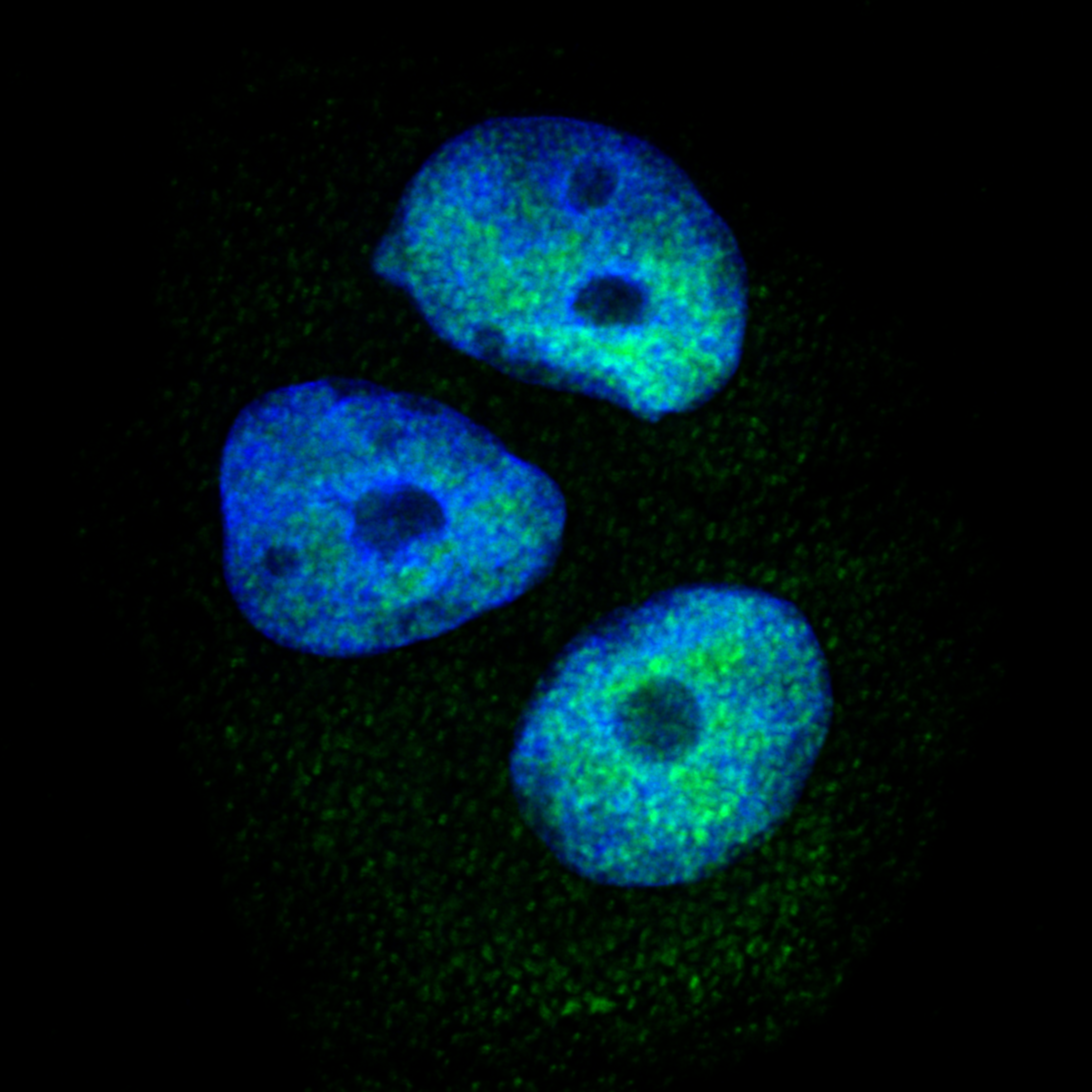

Supplement: Figure 2—figure supplement 1—source data 1. [file elife-87283-fig2-figsupp1-data1.zip › Figure 2-Figure supplement 1-source data/M3sg1-M3-Flag Merged.tif]

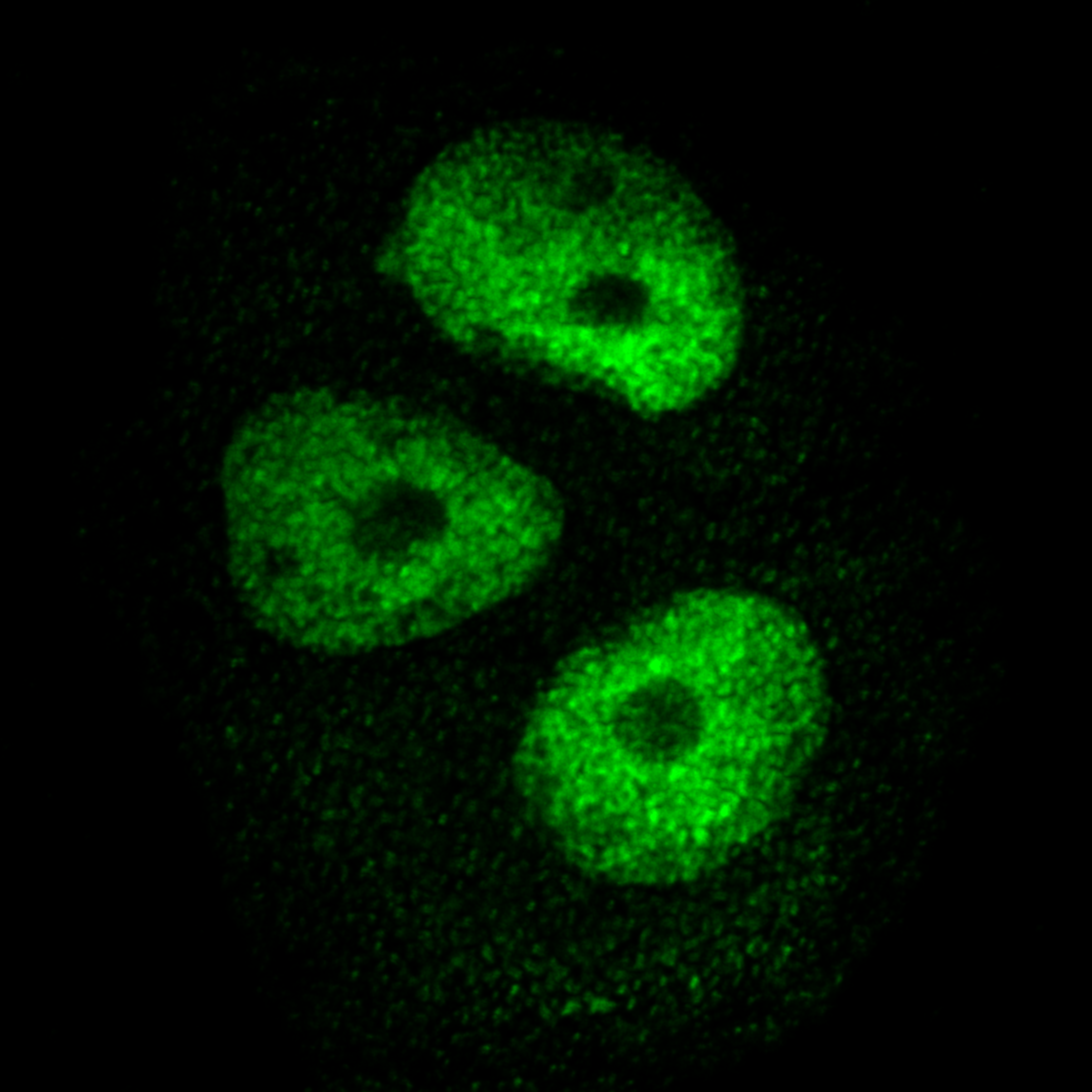

Supplement: Figure 2—figure supplement 1—source data 1. [file elife-87283-fig2-figsupp1-data1.zip › Figure 2-Figure supplement 1-source data/M3sg1-M3-Flag-488.tif]

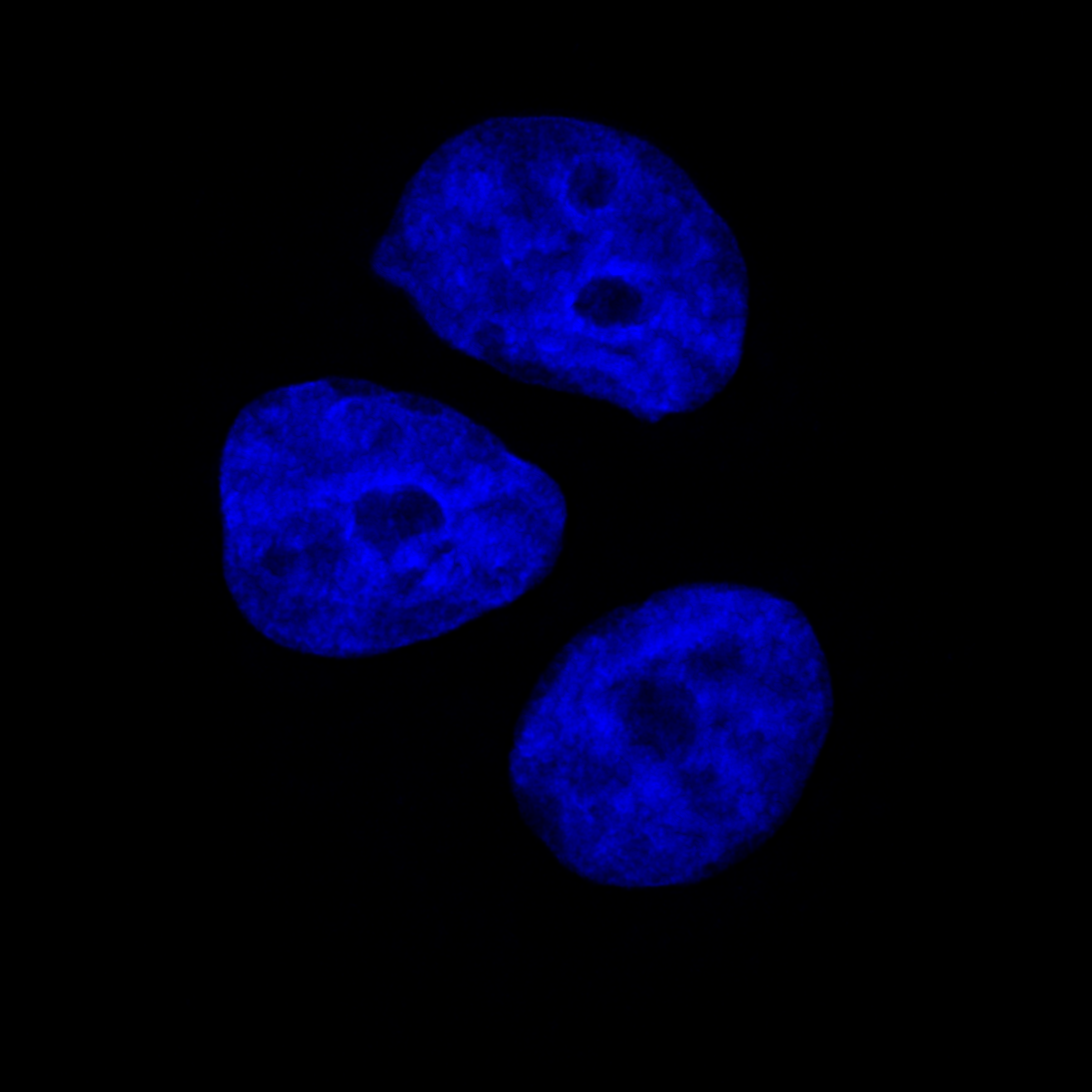

Supplement: Figure 2—figure supplement 1—source data 1. [file elife-87283-fig2-figsupp1-data1.zip › Figure 2-Figure supplement 1-source data/M3sg1-M3-Flag_DAPI.tif]

**Figure 3-source data-1:** Unedited western blot pictures for figure 3.

**a**

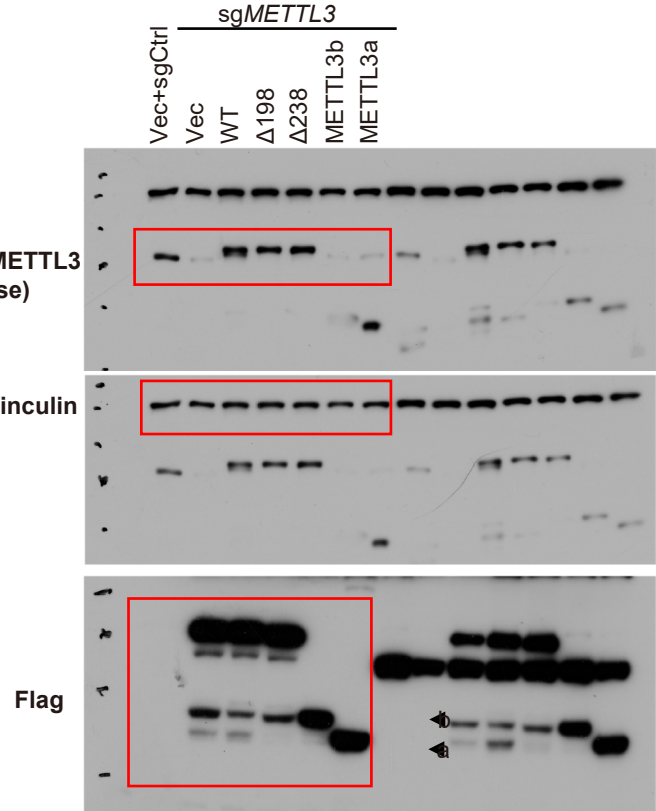

**c**

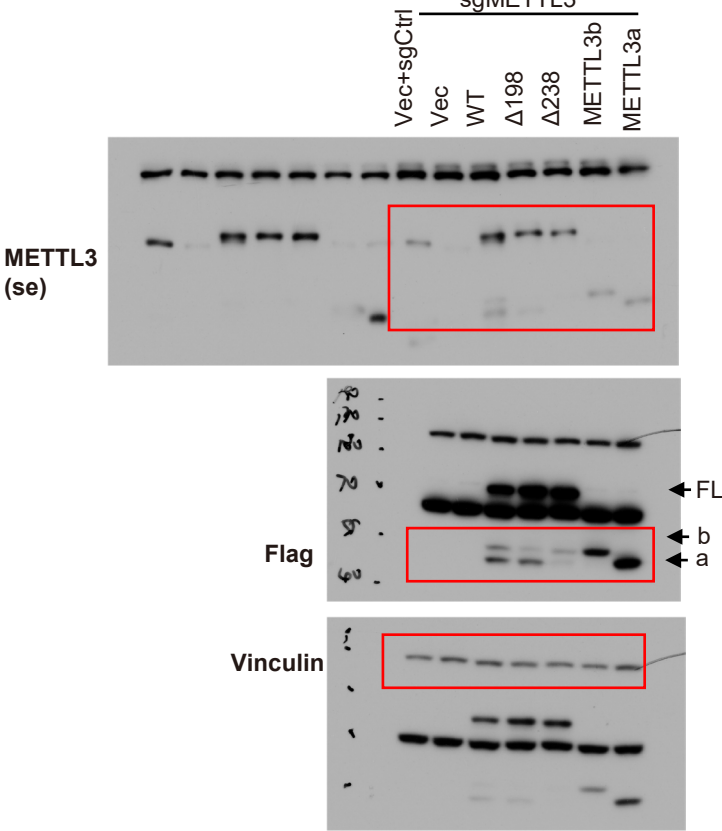

**e**

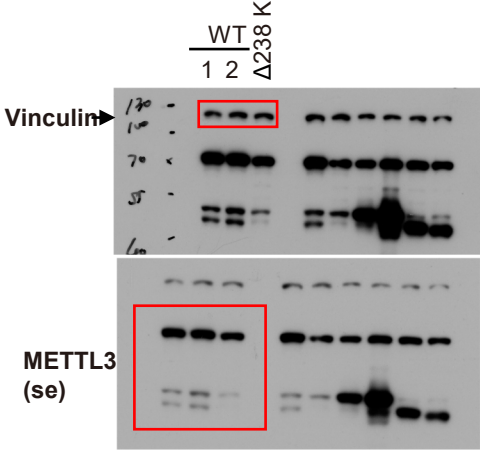

**g**

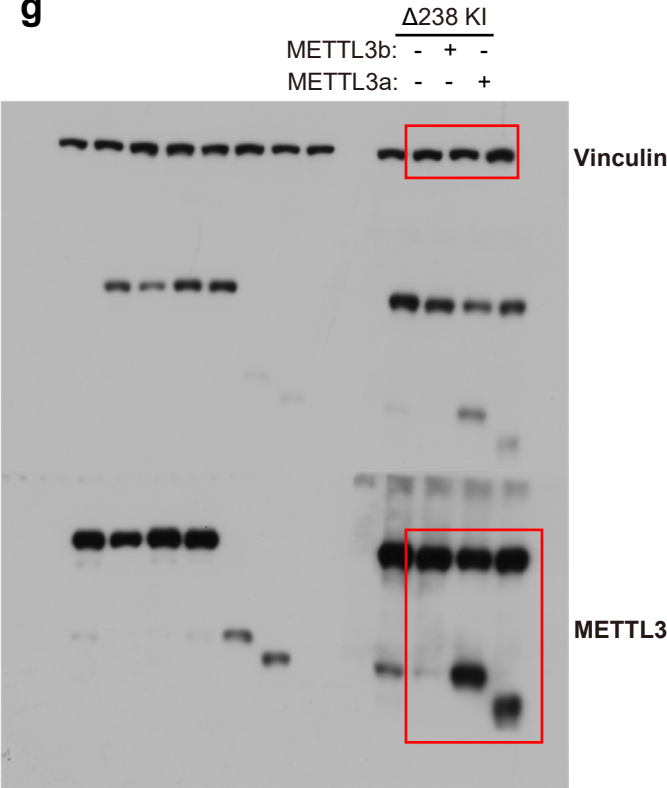

Supplement: Figure 3—source data 1. [file elife-87283-fig3-data1.pdf]

**Figure 4-source data:** Unedited western blot pictures for figure 4.

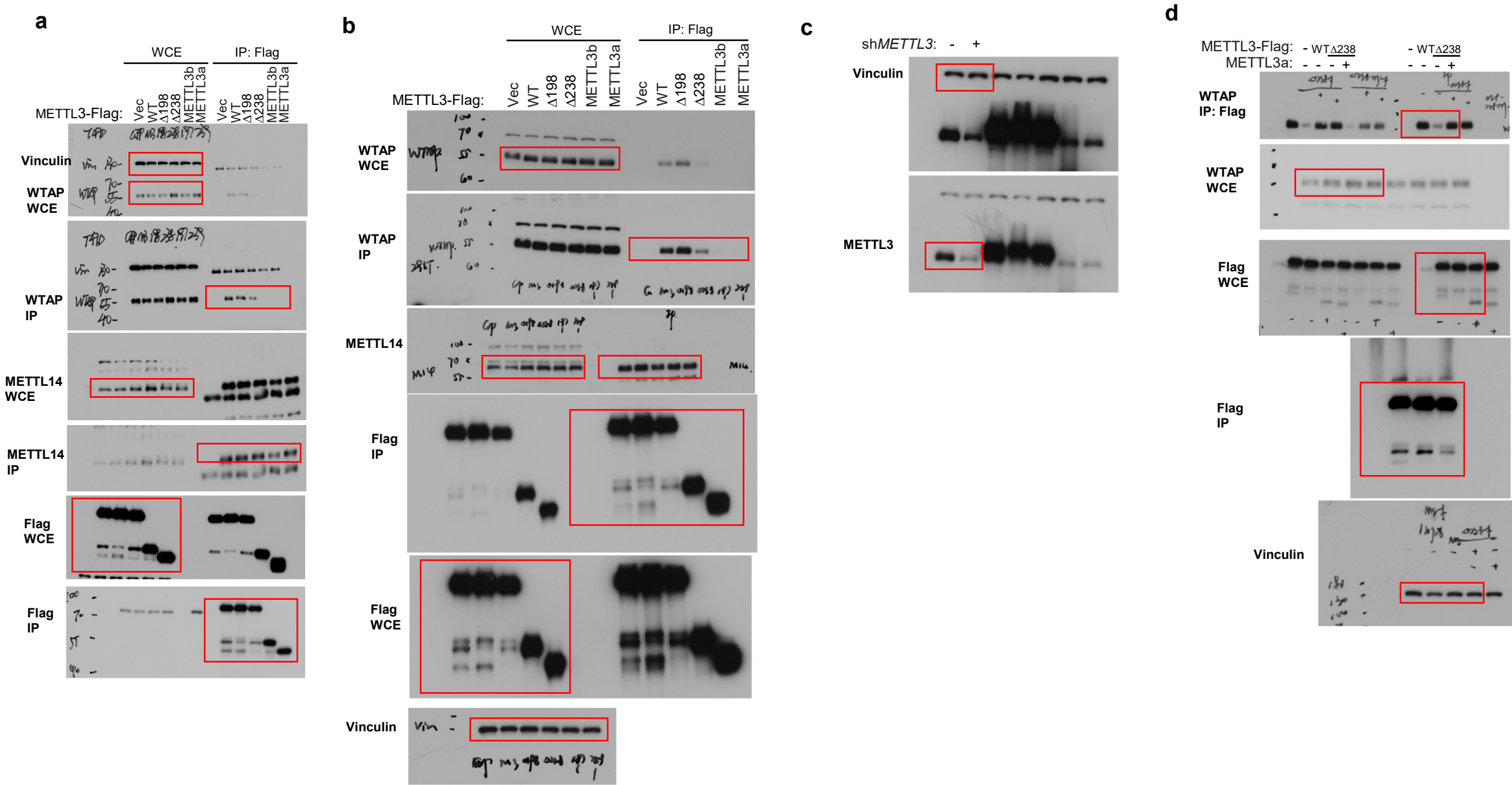

Supplement: Figure 4—source data 1. [file elife-87283-fig4-data1.pdf]

**Figure 6-source data-1:** Unedited western blot pictures for figure 6.

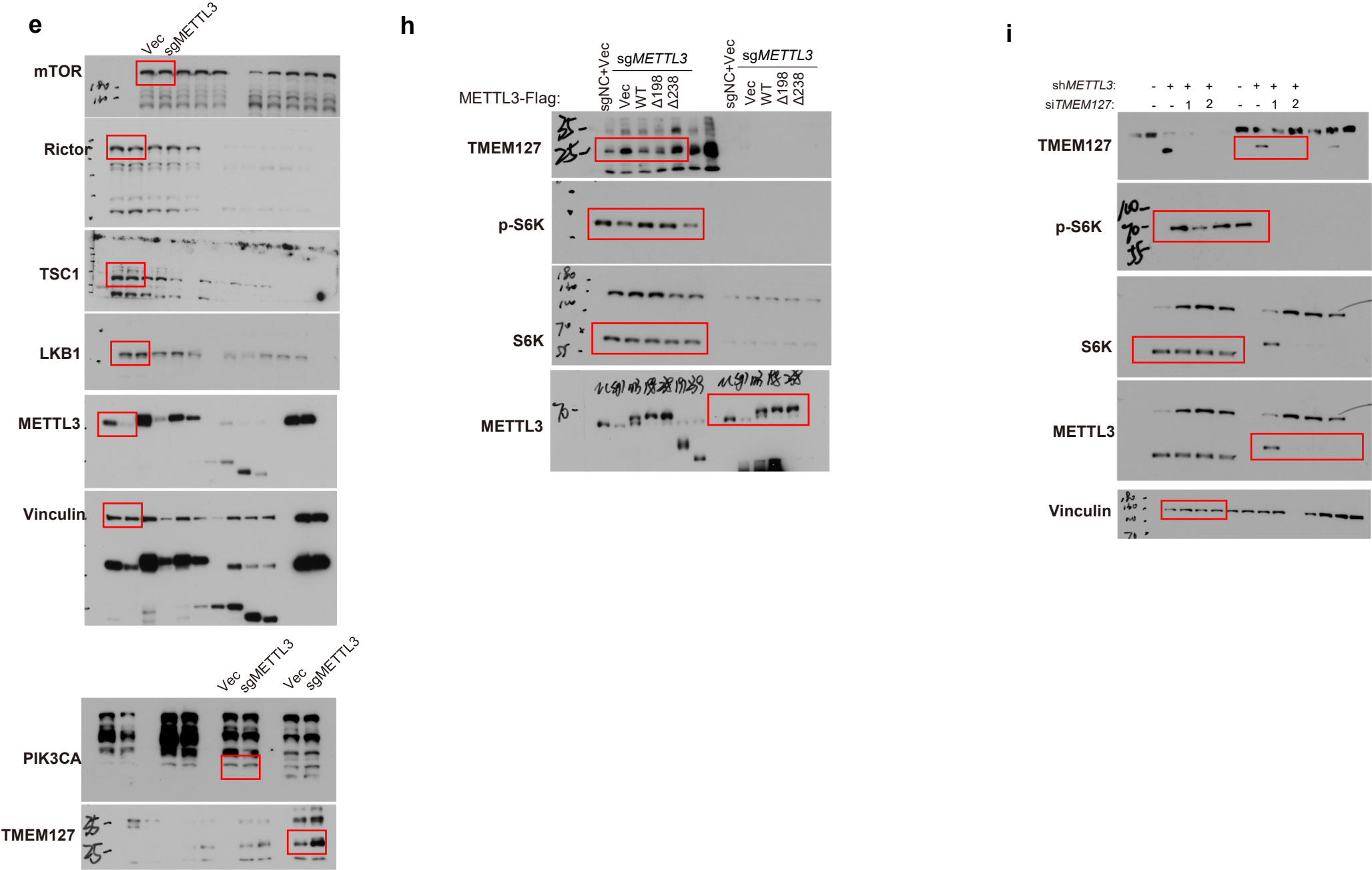

Supplement: Figure 6—source data 1. [file elife-87283-fig6-data1.pdf]

**Figure 6-Figure supplement 1:** Unedited western blot pictures for Figure 6-figure supplement 1.

**d**

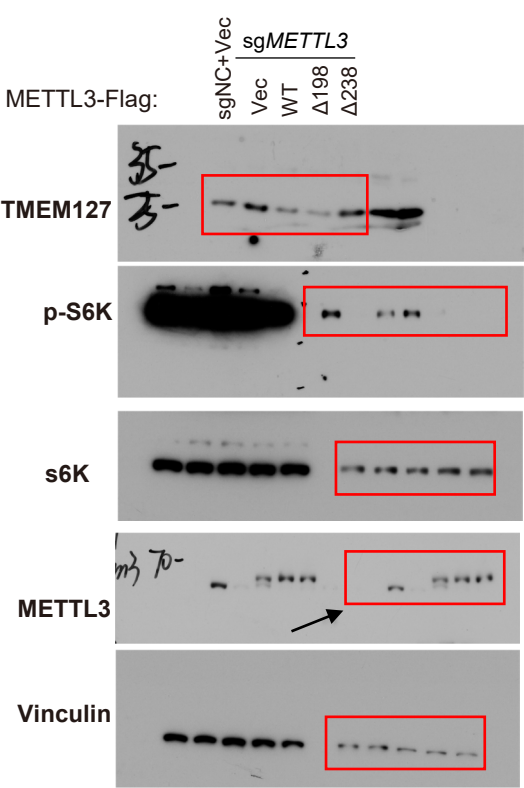

Supplement: Figure 6—figure supplement 1—source data 1. [file elife-87283-fig6-figsupp1-data1.pdf]

**Figure 7-source data:** Unedited western blot pictures for figure 7.

**a**

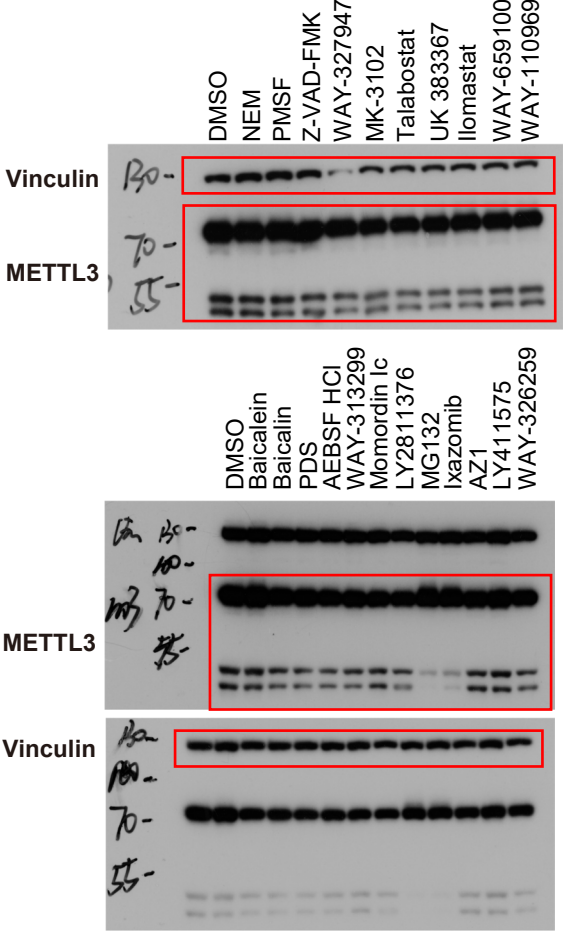

**b**

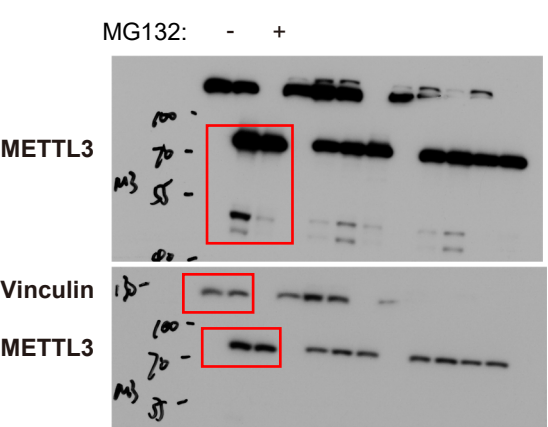

**c**

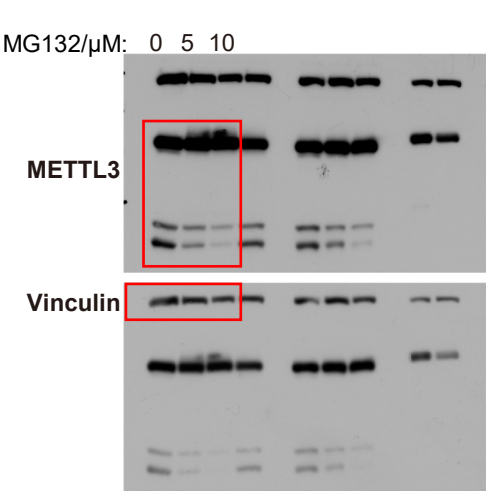

**d**

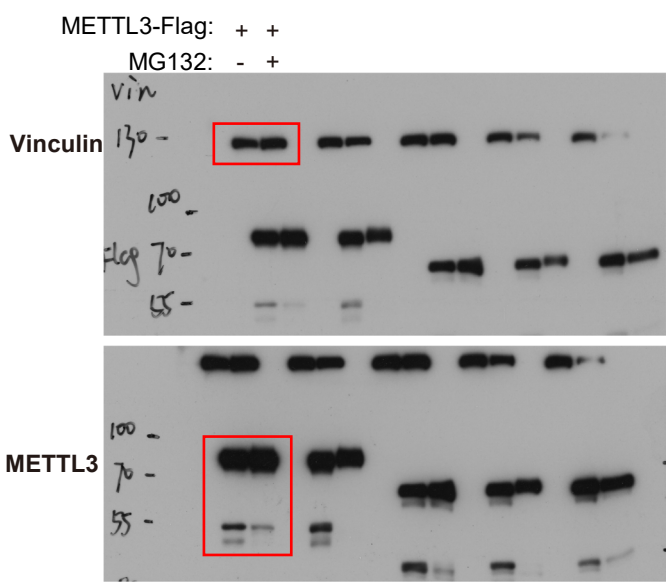

**e**

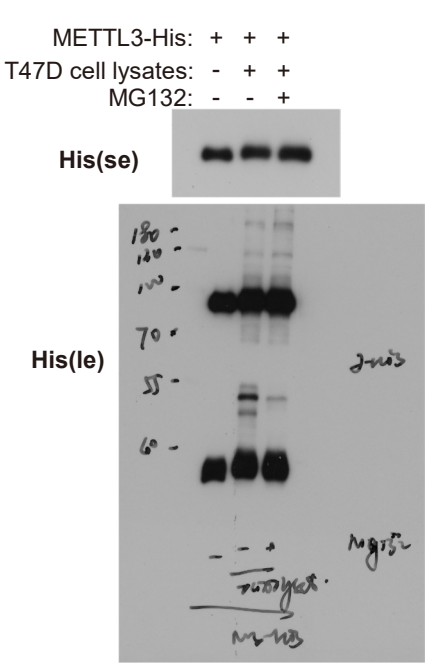

**g**

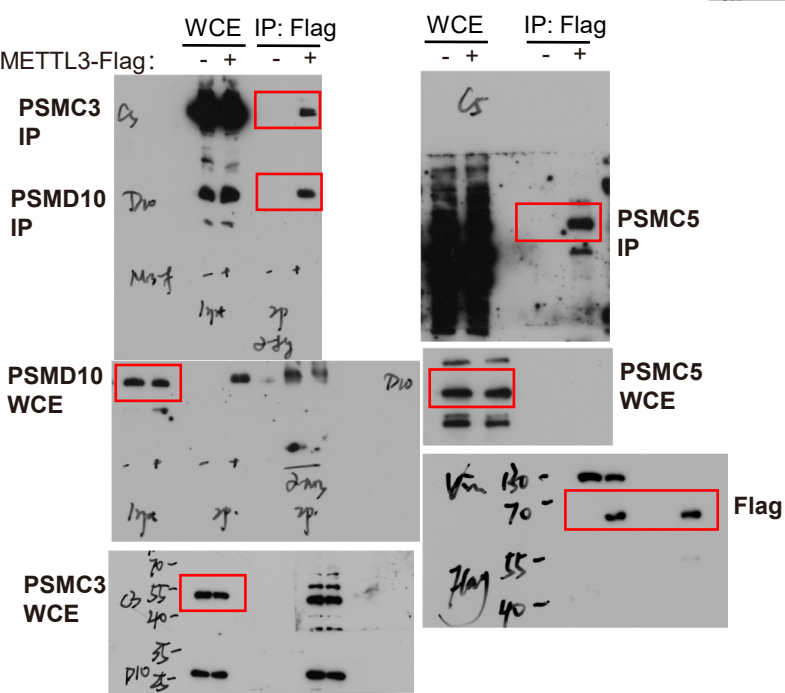

h

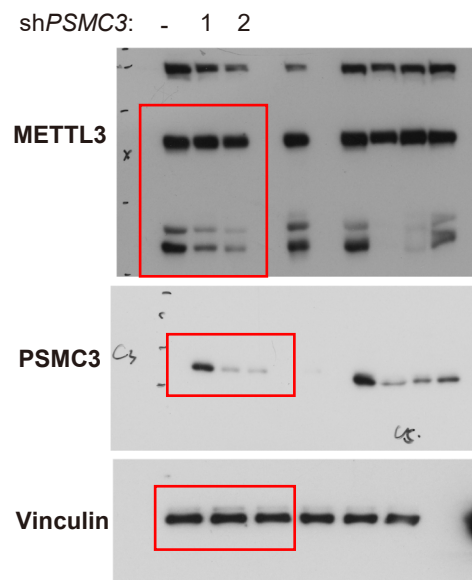

i

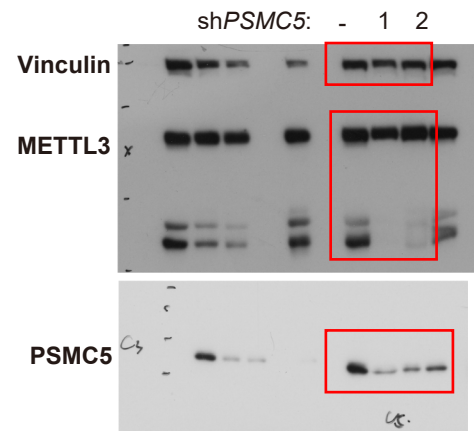

j

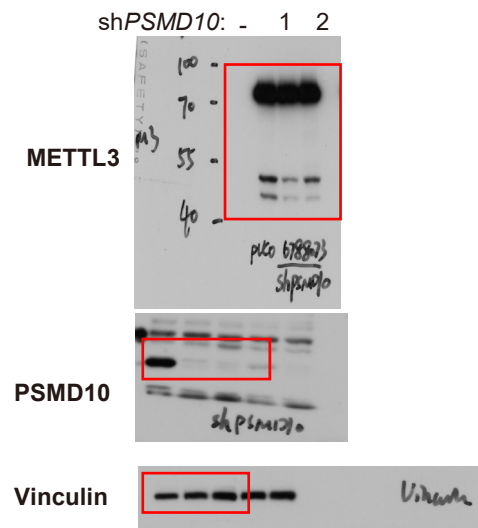

k

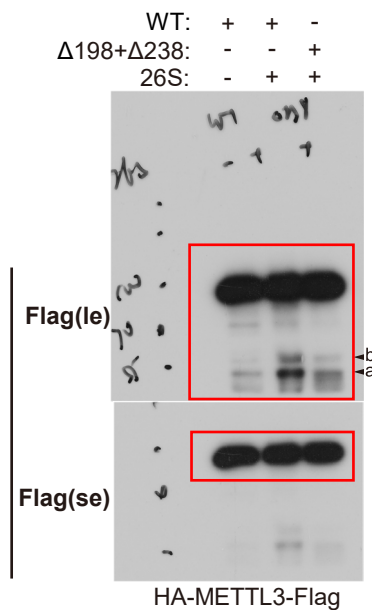

l

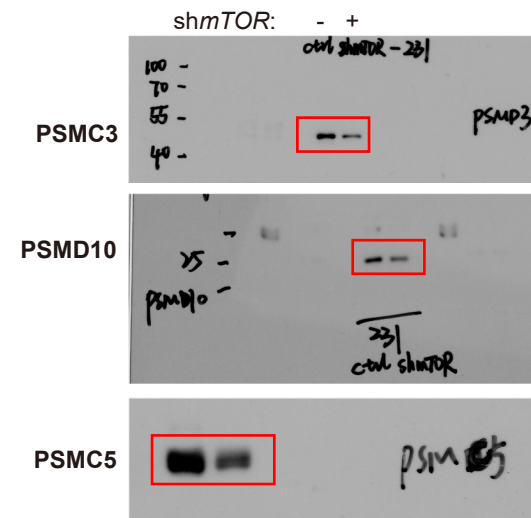

m

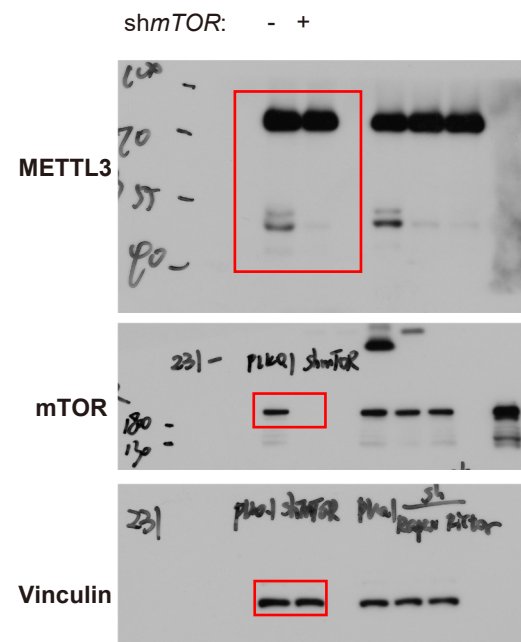

n

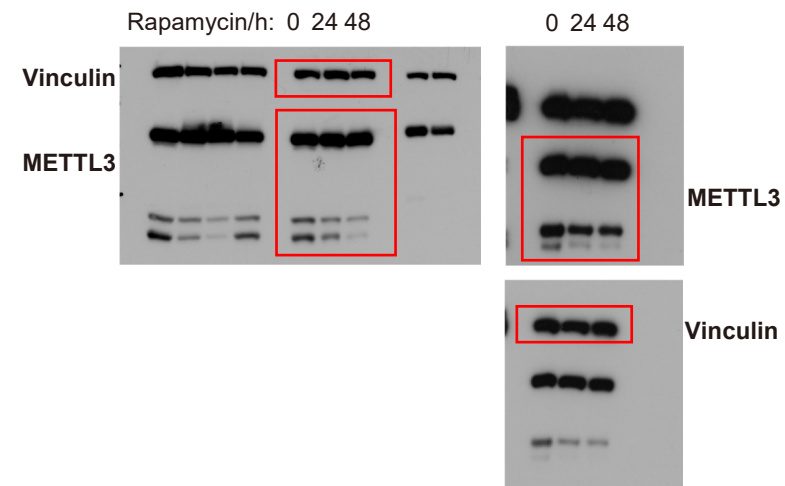

o

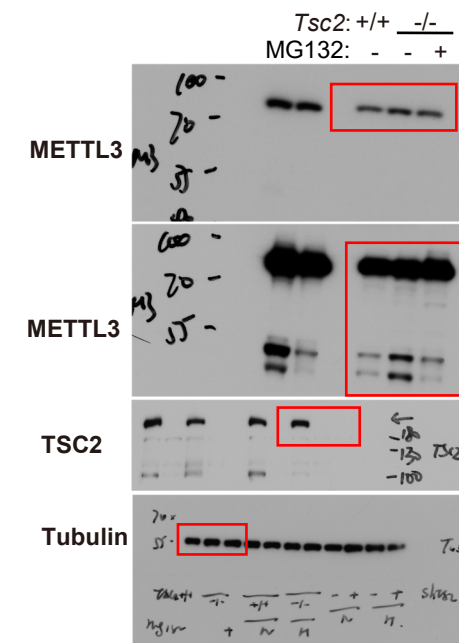

Supplement: Figure 7—source data 1. [file elife-87283-fig7-data1.pdf]

**Figure 7-Figure supplement 1:** Unedited western blot pictures for Figure 7-figure supplement 1.

**b**

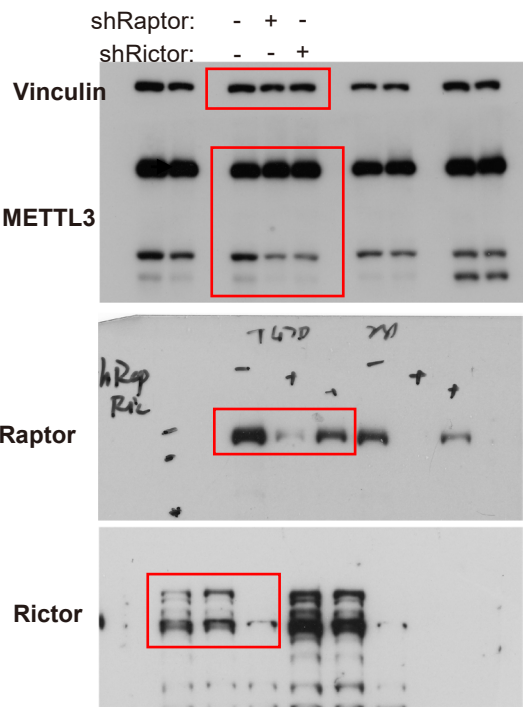

**c**

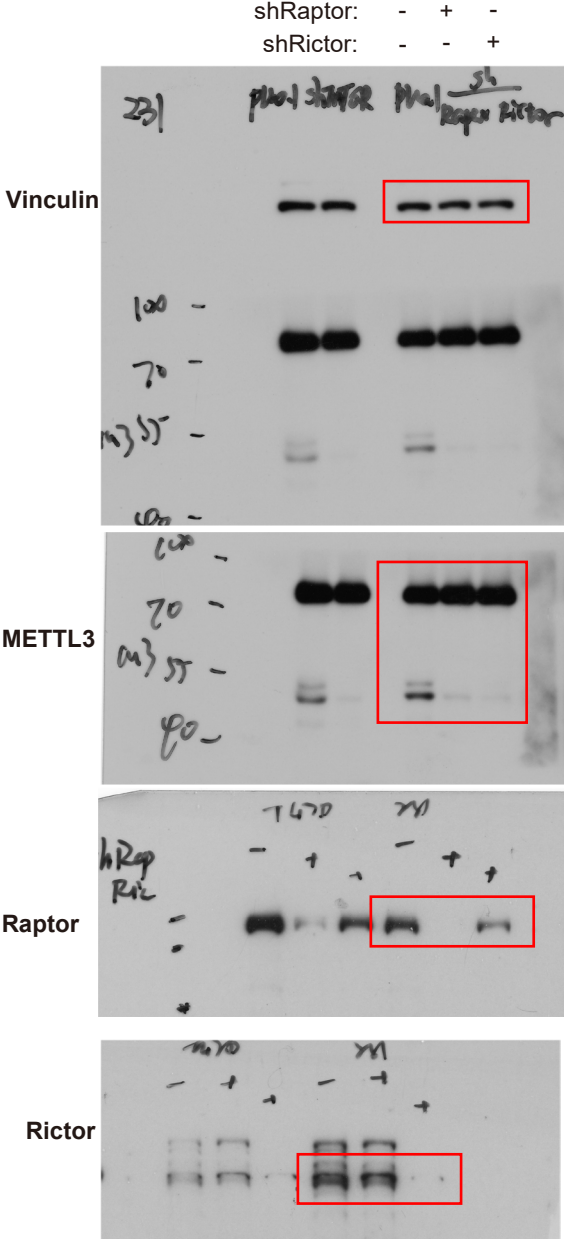

Supplement: Figure 7—figure supplement 1—source data 1. [file elife-87283-fig7-figsupp1-data1.pdf]
