# Supplementary material for: A cleaved METTL3 potentiates the METTL3–WTAP interaction and breast cancer progression
Source: eLife. 2023 Aug 17;12:RP87283. doi: 10.7554/eLife.87283 (PMC10435237; doi:10.7554/eLife.87283)

Figure 1- figure supplement 1-source data: Unedited western blot pictures for Figure 1-figure supplement 1.

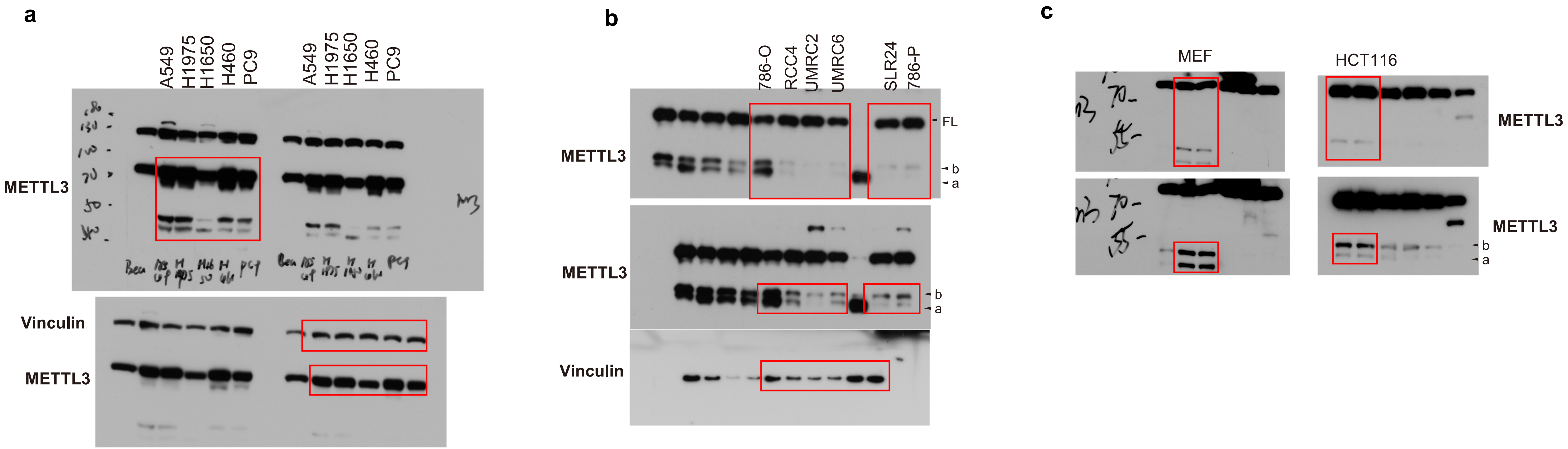

Supplement: Figure 1—figure supplement 1—source data 1. [file elife-87283-fig1-figsupp1-data1.pdf]
