# Supplementary material for: A cleaved METTL3 potentiates the METTL3–WTAP interaction and breast cancer progression
Source: eLife. 2023 Aug 17;12:RP87283. doi: 10.7554/eLife.87283 (PMC10435237; doi:10.7554/eLife.87283)
Supplement: Figure 5—source data 1. [file elife-87283-fig5-data1.pdf]

Figure 5-source data 1: Unedited western blot pictures for figure 5.

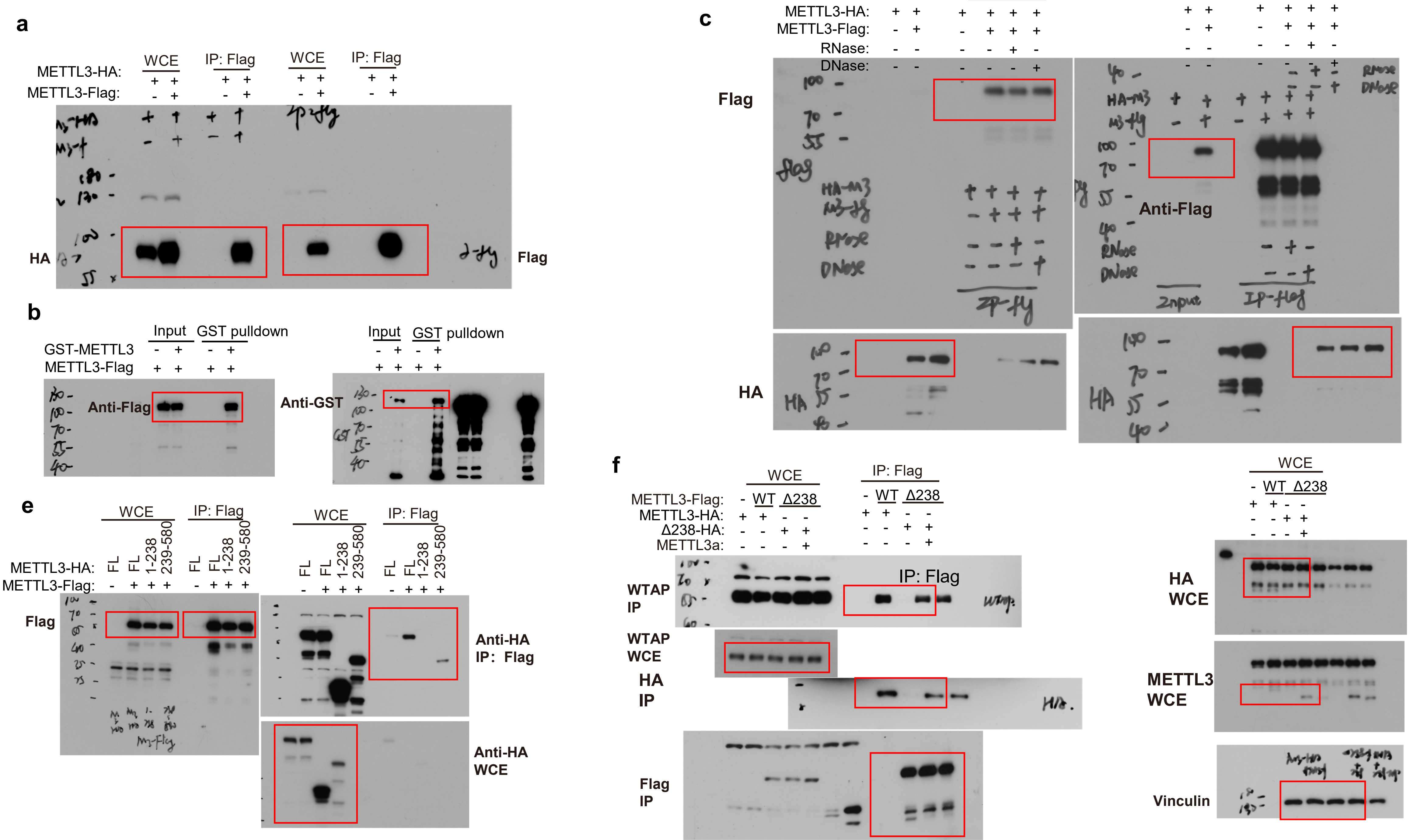

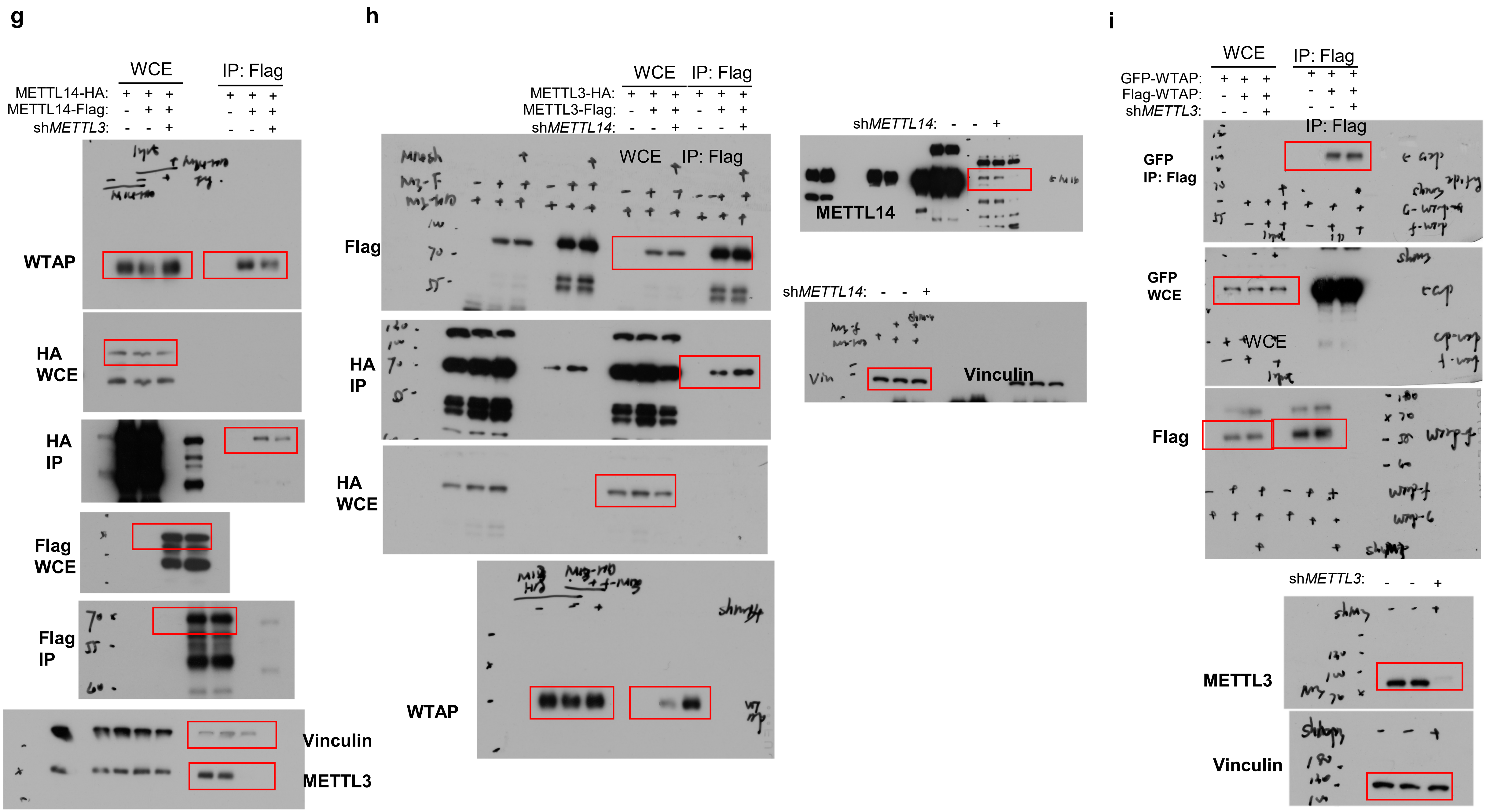
g
WCE
IP: Flag
METTL14-HA: + + + + + +
METTL14-Flag: - + + - + +
shMETTL3: - - + - - +
WTAP
HA WCE
HA IP
Flag WCE
Flag IP
Vinculin
METTL3
h
WCE
IP: Flag
METTL3-HA: + + + + + +
METTL3-Flag: - + + - + +
shMETTL14: - - + - - +
Flag
HA IP
HA WCE
WTAP
shMETTL14: - - +
METTL14
shMETTL14: - - +
Vinculin
i
WCE
IP: Flag
GFP-WTAP: + + + + + +
Flag-WTAP: - + + - + +
shMETTL3: - - + - - +
GFP IP: Flag
GFP WCE
Flag
shMETTL3: - - +
METTL3
Vinculin

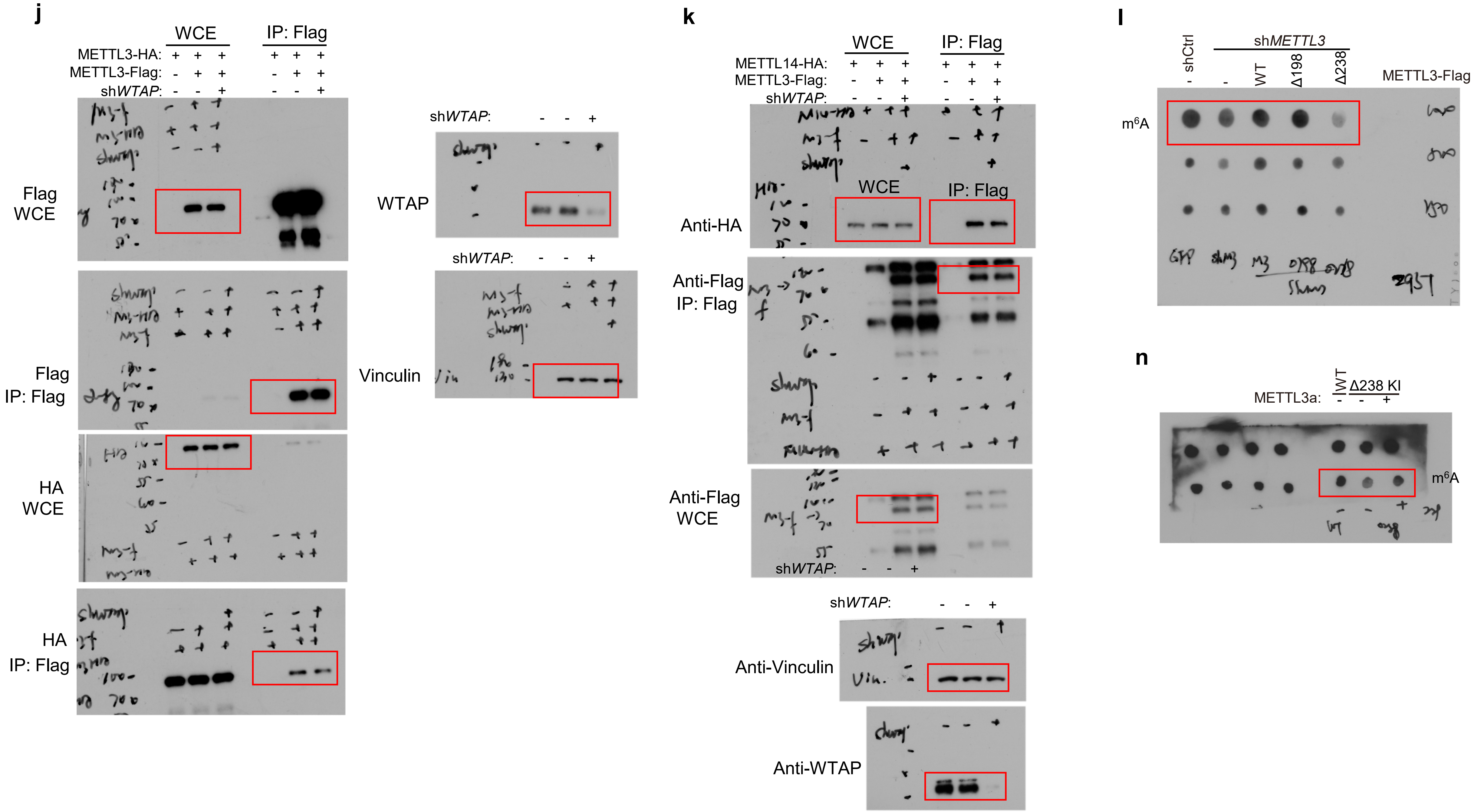

j
WCE
IP: Flag
METTL3-HA:
METTL3-Flag:
shWTAP:
Flag WCE
Flag IP: Flag
HA WCE
HA IP: Flag
WTAP
Vinculin
k
WCE
IP: Flag
METTL14-HA:
METTL3-Flag:
shWTAP:
Anti-HA
Anti-Flag IP: Flag
Anti-Flag WCE
Anti-Vinculin
Anti-WTAP
l
shCtrl
shMETTL3
WT
Δ198
Δ238
METTL3-Flag
m6A
n
METTL3a:
WT
Δ238 KI
m6A
